# Identify Key Genes Correlated to Ischemia-Reperfusion Injury in Aging Livers

**DOI:** 10.1155/2023/4352313

**Published:** 2023-02-16

**Authors:** Xijing Yan, Jinliang Liang, Xuejiao Li, Zhongying Hu, Jiahao Chen, Jun Zheng, Rong Li

**Affiliations:** ^1^Department of Hepatic Surgery and Liver Transplantation Center, Third Affiliated Hospital of Sun Yat-Sen University, Guangzhou 510630, China; ^2^Guangdong Provincial Key Laboratory of Liver Disease Research, Third Affiliated Hospital of Sun Yat-Sen University, Guangzhou 510630, China; ^3^Department of Laboratory Medicine, Third Affiliated Hospital of Sun Yat-Sen University, Guangzhou 510630, China

## Abstract

**Background:**

With the intensification of population aging, the proportion of aging livers in the donor pool is increasing rapidly. Compared with young livers, aging livers are more susceptible to ischemia-reperfusion injury (IRI) during liver transplantation, which greatly affects the utilization rate of aging livers. The potential risk factors associated with IRI in aging livers have not been fully elucidated.

**Methods:**

In this work, five human liver tissue expression profiling datasets (GSE61260, GSE107037, GSE89632, GSE133815, and GSE151648) and a total of 28 young and aging liver tissues of human (*N* = 20) and mouse (*N* = 8) were used to screen and verify the potential risk factors associated with aging livers being more prone to IRI. DrugBank Online was used to screen drugs with potential to alleviate IRI in aging livers.

**Results:**

The gene expression profile and immune cell composition between young and aging livers had significant differences. Among the differentially expressed genes, aryl hydrocarbon receptor nuclear translocator-like (ARNTL), BTG antiproliferation factor 2 (BTG2), C-X-C motif chemokine ligand 10 (CXCL10), chitinase 3-like 1 (CHI3L1), immediate early response 3 (IER3), Fos proto-oncogene, AP-1 transcription factor subunit (FOS), and peroxisome proliferative activated receptor, gamma, coactivator 1 alpha (PPARGC1A), mainly involved in the regulation of cell proliferation, metabolism, and inflammation, were also dysregulated in liver tissues suffered from IRI and could form a FOS-centered interaction network. Nadroparin was screened out with the potential to target FOS in DrugBank Online. In addition, the proportion of dendritic cells (DCs) was significantly upregulated in aging livers.

**Conclusions:**

We combined the expression profiling datasets of liver tissues and samples collected in our hospital for the first time to reveal that the changes in the expression of ARNTL, BTG2, CXCL10, CHI3L1, IER3, FOS, and PPARGC1A and the proportion of dendritic cells may be associated with aging livers being more prone to IRI. Nadroparin may be used to mitigate IRI in aging livers by targeting FOS, and regulation of DC activity may also reduce IRI.

## 1. Introduction

Liver transplantation is the only effective treatment for the end-stage liver disease, such as liver cancer, acute liver failure, and certain metabolic liver disease. However, the shortage of liver donors severely restricts the development of liver transplantation. To solve that problem, the use of marginal liver grafts has become an inevitable choice. With the intensification of population aging, the proportion of elderly donor livers in the marginal donor pool is increasing. There is no criteria age limit for aging liver; we usually default the livers derived from donors over 50 years as aging livers [1]. According to statistics, in 2016, the proportion of elderly donor livers in the donor pool of the United States of America (USA) was about 35%, and this proportion was as high as 60% in France [2]. Improving the utilization of elderly donor livers in transplantation is essential to alleviate the problem of donor shortage.

The synthetic capability of the liver is minimally affected by age due to its good reserve function and dual blood supply unlike the heart or the kidney. However, compared with young livers, aging livers have three prominent problems: (1) limited liver regeneration ability, (2) more prone to ischemia reperfusion injury (IRI), and (3) more susceptible to liver tumor or hepatitis B virus (HBV)/hepatitis C virus (HCV) recurrence with graft fibrosis and cirrhosis [3]. The graft undergone ischemia-reperfusion (IR) is inevitable during liver transplantation. IRI is one of the key factors which induce graft loss, posttransplant complications, and early liver failure [4]. The reasons why aging livers are more prone to IRI in transplantation are multifaceted and extremely complex which have not been fully clarified until now. It is urgent to further identify the potential risk factors correlated to aging livers more prone to IRI and develop new methods to reduce IRI of aging livers in transplantation.

In this study, four expression profiling datasets of human liver tissues (GSE61260, GSE107037, GSE89632, and GSE133815) were used to analyze the differences in gene expression and immune cell composition between young and aging livers. A high-throughput sequencing dataset (GSE151648) was used to screen the differential genes associated with liver IRI in transplantation. Then, the correlation analysis between aging livers and IRI was performed based on the above differential genes. A series of genes mainly involved in the regulation of cell proliferation, metabolism, and inflammation, such as aryl hydrocarbon receptor nuclear translocator-like (ARNTL), BTG antiproliferation factor 2 (BTG2), C-X-C motif chemokine ligand 10 (CXCL10), chitinase 3-like 1 (CHI3L1), immediate early response 3 (IER3), Fos proto-oncogene, AP-1 transcription factor subunit (FOS), and peroxisome proliferative activated receptor, gamma, coactivator 1 alpha (PPARGC1A), were identified with the similar expression trend in both aging livers and livers suffered from IRI and formed a FOS-centered interaction network. In addition, we also found that dendritic cells (DCs) were significantly upregulated in aging livers. These changes in aging livers were further validated with liver specimens collected in our center. Then, we used DrugBank Online to screen out nadroparin which can target FOS, with the potential to mitigate IRI in aging livers. Taken together, our findings suggested that the changes in the expression of ARNTL, BTG2, CXCL10, CHI3L1, IER3, FOS, and PPARGC1A and the proportion of DC may account for the susceptibility of aging livers to IRI, which provided new targets and theoretical basis for developing new methods to alleviate IRI in aging livers.

## 2. Materials and Methods

### 2.1. Data Sources

Five human liver tissue expression profiling datasets, GSE61260, GSE107037, GSE89632, GSE133815, and GSE151648, were used in this work. GSE61260 is an array-based mRNA expression profiling for human liver tissues (*N* = 134) obtained from morbidly obese patients and healthy controls, ages varied from 10 to 85. Only sequencing data from healthy controls (*N* = 66) were used for further analysis. GSE107037 is a gene expression profiling of liver biopsies collected from 33 heathy donors ranging from 13 to 90 years old. GSE89632 is a gene expression profiling of hepatic gene expression for patients with nonalcoholic fatty liver disease (*N* = 39) and healthy controls (*N* = 24) ranging from 22 to 68, and only sequencing data from healthy controls (*N* = 24) were used for further analysis. GSE133825 is a gene expression profiling of liver samples for young (*N* = 11) and old (*N* = 12) men and women to evaluate changes in the expression of xenobiotic metabolism enzymes and transporters. When grouping the samples in the above data sets, we defined age = 50 as the cut off. GSE151648 is a high-throughput sequencing for needle-core liver biopsies obtained from orthotopic liver transplants (*N* = 40). Samples were collected at 2 time points: pretransplantation (*N* = 40) and posttransplantation (*N* = 40). Among all 40 recipients, 23 were suffered from IRI and 17 were with no IRI. Only sequencing data of recipients with IRI were used for further analysis. All these data are publicly available and downloaded from NCBI GEO DataSets (https://www.ncbi.nlm.nih.gov/gds/?term=).

### 2.2. Tissue Specimens

A total of 20 liver tissues derived from donors in liver transplantation were used in this study, and all donors had no liver-related disease. The livers derived from donor≧50 years were defined as aging livers (*n* = 10), and the livers derived from donor < 50 years were defined as young livers (*n* = 10). All procedures with these specimens in this study were conducted in accordance with the Institutional Research Ethics Committee of the third affiliated hospital, Sun Yat-Sen University (Guangzhou, Guangdong, China). Informed written consent was obtained from all participants as well. Four 6-month-old C57 mice (young mice) and four 24-month-old C57 mice (old) were used in this study, and all experimental procedures involving animals were conducted in accordance with the Chinese legislation regarding experimental animals and were approved by the Animal Ethical and Welfare Committee of the Third Affiliated Hospital, Sun Yat-Sen University (Guangzhou, Guangdong, China).

### 2.3. Functional Enrichment Analysis

Based on the differential genes between young and aging livers, the functional enrichment analysis was conducted using clusterProfiler and ReactomePA packages in R and displayed in bubble charts as previously described. The size of the bubble showed the number of differential genes, and the color of the bubble showed the *P* value. *P* < 0.05 was considered statistically significant.

### 2.4. Immune and Stromal Cell Composition Analysis

Immune and stromal cell composition analysis was conducted using xCell package in R and displayed in heatmaps. The differential cell types between young and aging livers were further identified with Student's *t*-test. *P* < 0.05 was considered statistically significant.

### 2.5. The Correlation Heatmap Analysis

To visually display the correlation between the differential genes and aDC, the correlation analysis was conducted using corrplot package in R and displayed in the correlation heatmaps. In correlation heatmaps, blue showed positive correlation, and red showed negative correlation; moreover, the darker the color, the larger the bubble, the higher the correlation.

### 2.6. Immunohistochemistry (IHC)

IHC was conducted according to a standard method previously described [5]. In brief, paraffin-embedded specimen sections were deparaffinized with xylenes and rehydrated. Then, the sections were performed ethylenediaminetetraacetic acid (EDTA) antigenic retrieval. After the sections returned to room temperature, 3% hydrogen peroxide in methanol was used to quench the endogenous peroxidase activity in sections, followed by incubation with 1% bovine serum albumin to block nonspecific binding. Anti-CD11c (1 : 300; Cell Signaling Technology, Danvers, MA, USA) was incubated with the sections overnight at 4°C. The corresponding results of IHC were photographed with upright fluorescence microscope (DM3000, Leica, Germany).

### 2.7. RNA Extraction and qRT-PCR

Total RNA was extracted from human liver tissues using TRIzol reagent (Invitrogen, Carlsbad, CA, USA) according to the manufacturer's instructions. The concentration and purity of RNA were measured using a NanoDrop 2000 apparatus (Thermo Fisher Scientific, Waltham, MA, USA). The RNA integrity was evaluated by the ratio of 28S : 18S after denaturing formaldehyde agarose gel electrophoresis. cDNA was synthesized with Evo M-MLV RT Kit with gDNA Clean for qPCR II (Accurate Biology, Hunan, China) according to the manufacturer's instructions. qRT-PCR was performed using a LC480 real-time PCR detection system (Roche, Basel, Switzerland) with GoTaq qPCR Master Mix (Promega, Madison, Wisconsin, USA). All primers used for qRT-PCR were designed using Beacon Designer 7.0 and synthesized by BGI Tech (Shenzhen, China). Glyceraldehyde-3-phosphate dehydrogenase (GAPDH) served as internal control. The sequences of all primers used are listed in Supplementary table 3.

### 2.8. Statistical Methods

LIMMA package in R and GraphPad Prism 8.0 software package (GraphPad Software, Inc., San Diego, CA, USA) were used to analyze the gene expression in different types of specimens. The differences in transcriptional expression were conducted using Student's *t*-test. *P* values < 0.05 were considered statistically significant.

## 3. Results

### 3.1. Significant Differences in the Gene Expression Profile between Young and Aging Livers

**Figure 1:**
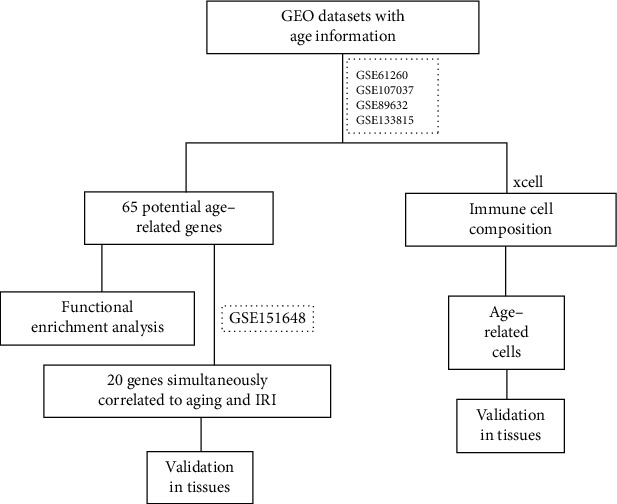
Flow chart of our research.

**Figure 2:**
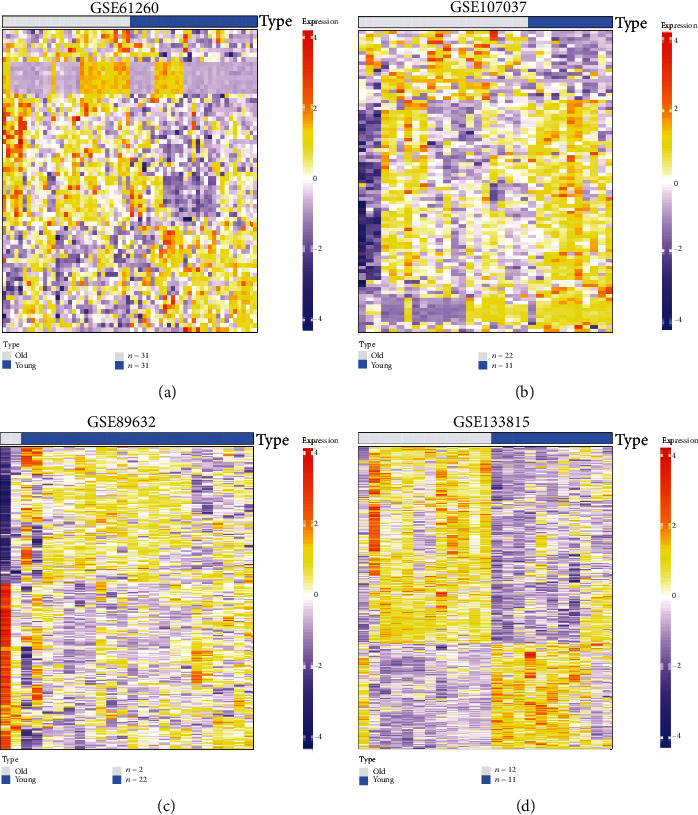
The gene expression profiles of young and aging livers. Heatmaps were used to intuitively display the differential gene expression profiles between young and aging livers of GSE61260 (a), GSE107037 (b), GSE89632 (c), and GSE133815 (d). All gene expression values were normalized with log_2_^Value^, and the criteria for defining differential genes between young and aging livers were as follows: |log fold change| ≥ 0.5 and *P* < 0.05.

We conducted our research to identify the risk factors correlated to IRI in aging livers according to the flowchart showed in Figure 1. The differential genes between young and aging livers may affect the susceptibility of IRI in transplantation; 4 gene expression profiling datasets of human liver tissues (GSE61260, GSE107037, GSE89632, and GSE133815) downloaded from NCBI GEO DataSets (https://www.ncbi.nlm.nih.gov/gds) were used to initially screen the differential genes. The samples with no history of liver-related diseases were extracted from the above 4 expression profiling datasets to do further analysis. We divided the selected samples of each dataset into two groups, young (age < 50) and old (age≧50). As shown in Figures 2(a)–2(d), heatmaps were used to display the gene expression profiles of young and aging livers. As we expected, despite the individual differences between people, there were still significant differences in the gene expression profile between young and aging livers. In order to more clearly show the numbers of differential genes in each gene set and their related up-/downregulation profiles, we also drew the corresponding volcano maps (Supplementary Figure 1). The lists of differential genes in each gene set are shown in supplementary table 1.

### 3.2. Functional Enrichment Analysis of Differential Genes

**Figure 3:**
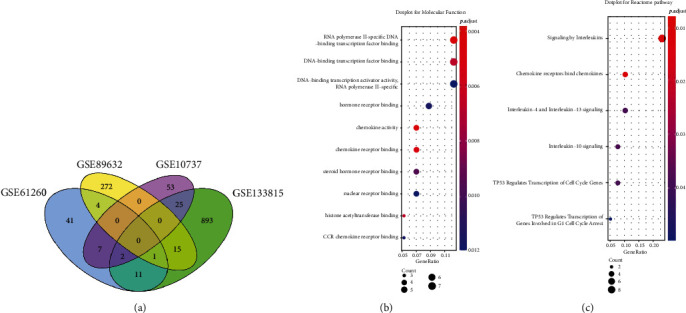
The functional enrichment analysis of differential genes between young and aging livers. (a) Venn diagram showed the number of differential genes in GSE61260, GSE107037, GSE89632, and GSE133815. (b) Bubble chart showed the enriched molecular functions (MF) correlated to the differential genes. (c) Bubble chart showed the related signaling pathways involved by the differential genes. The size of the bulb represented the number of genes involved, and the color of the bulb represented the *P* value. *P* < 0.05 was considered statistically significant.

Given that the differences in sequencing platforms, the genes with |log fold change| ≥ 0.5 and *P* < 0.05 in two or more gene expression profiling datasets at the same time were defaulted as differential genes. A total of 65 genes were eventually identified as differential genes and were displayed with Venn diagram (Figure 3(a)). Then, these genes were further performed molecular function analysis and Reactome pathway analysis. As shown in Figure 3(b), the molecular function analysis result suggested that the differential genes were mainly involved in the following biological process: RNA polymerase II-specific DNA-binding transcription factor binding, hormone receptor binding, chemokine activity and receptor binding, nuclear receptor binding, and histone acetyltransferase bind, which implied that the activities of gene replication, transcription, proliferation, metabolism, and immunity were changed between young and aging livers. The Reactome Pathway Analysis results (Figure 3(c)) showed that the differential genes were mainly participated in the regulation of the following signaling pathways: signaling by interleukins, chemokine receptors bind chemokines and tumor protein p53 (TP53) regulated transcription of cell cycle genes. These signaling pathways further suggested that the activities of cell proliferation, metabolism, and immunity were changed in aging livers.

### 3.3. Changes in Immune Cell Composition of Young and Old Livers

**Figure 4:**
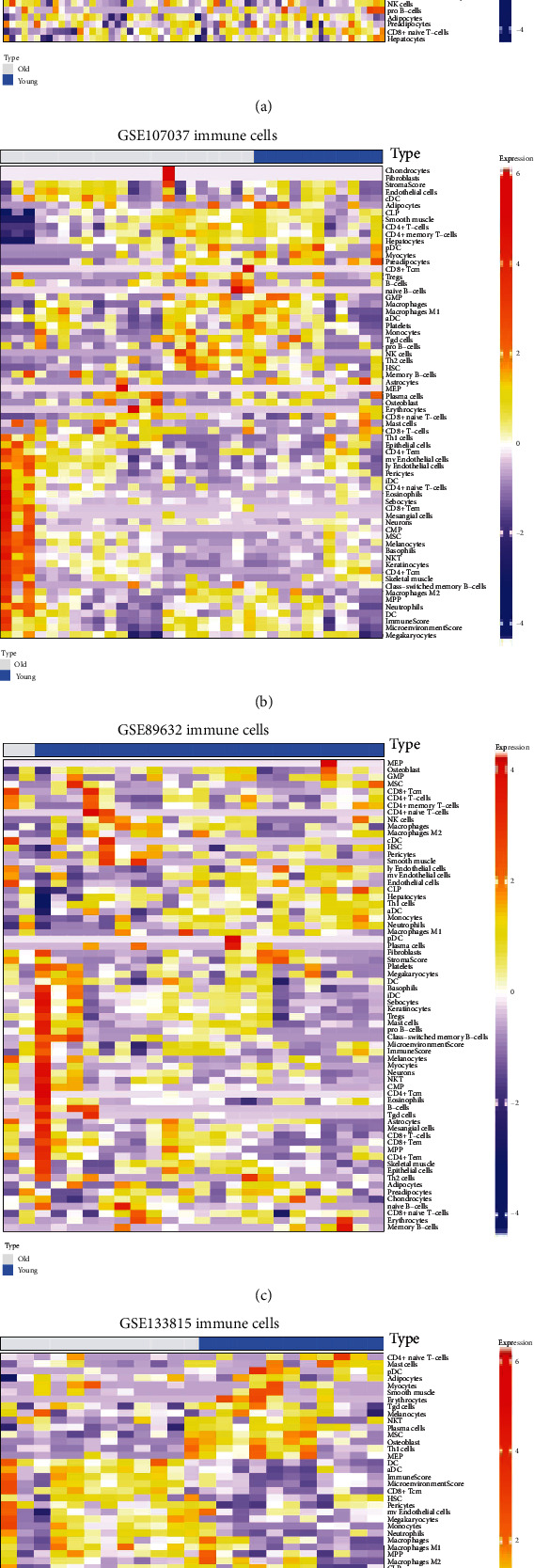
The composition of immune cells and stromal cells in young and aging livers. Based on the expression profiling datasets, xCell was used to analyze the composition of 64 common immune and stromal cells in young and aging livers. The heatmaps were used to display the detail cell composition between young and aging livers of GSE61260 (a), GSE107037 (b), GSE89632 (c), and GSE133815 (d).

Compared with young livers, grafts from old donors are more susceptible to IRI. The previous studies proved that an intense inflammatory process occurs during IRI [6]. The functional enrichment analysis results in Figure 3 suggested that there were significant changes in the activities of immunity between young and aging livers. Therefore, we speculated that these changes may influence the susceptibility of aging livers to IRI. Given that immune cells play pivotal roles in the inflammatory response, we further analyzed the immune cell composition of young and aging livers. The heatmaps in Figures 4(a)–4(d) intuitively showed the composition profiles of 64 common types of stroma and immune cells in young and aging livers. We found that there were significant individual differences in the composition of immune cells within human liver. In addition to individual differences, immune cell composition also changed with age, especially showed in the heatmap of GSE133815 (Figure 4(d)). The differential immune cell populations in young and old liver tissues may contribute to the susceptibility of aging livers to IRI.

### 3.4. Activated Dendritic Cells (aDC) Were Upregulated in Aging Livers and Positively Correlated with the Expression of Some Proinflammatory Cytokines

**Figure 5:**
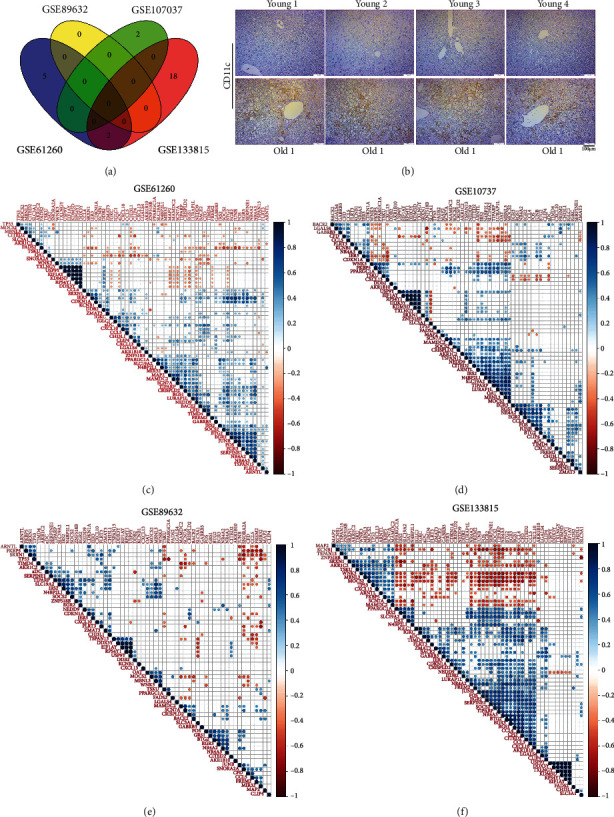
Screening of differential immune cells and analysis of correlated genes. (a) Venn diagram showed the number of differential immune cells in GSE61260, GSE107037, GSE89632, and GSE133815. The cells appeared in two or more datasets were selected for further analysis. (b) Representative results of immunohistochemical staining for CD11c, a typical marker for DCs, in young and aging liver tissues. Scale bars, 100 *μ*m. (c–f) The correlation analysis between DCs and the differential genes between young and aging livers were performed and displayed as correlation heatmaps.

Student's *t*-test was performed to screen which cell types making differences between young and aging livers. The detail analysis results are shown in supplementary table 2. The cell types with significantly different proportion (*P* < 0.05) between young and aging livers in two or more datasets simultaneously were selected for further analysis. As shown in Figure 5(a), two types of cells finally attracted our attention, namely, activated dendritic cells (aDC) and sebocytes. Because the proportion of sebocytes in the livers is extremely low (approximate 0% in supplementary table 1) and it was never been reported in the livers before, we cannot be sure whether sebocytes exist in the livers and no further researches were performed on it. Another type of cells, aDC, was significantly upregulated in aging livers (supplementary table 1). We collected liver tissues derived from 24-month-old and 6-month-old C57 mice, and immunohistochemistry was used to detect the proportion of DCs in the liver tissues. Consistent with the results obtained by biosynthesis analysis, the proportion of DCs in aging livers was significantly higher than that in young livers (Figure 5(b)). To reveal the potential function of aDC in aging livers, we further analyzed the correlation between aDC and the differential genes mentioned in Figure 3. The correlation analysis results (Figures 5(c)–5(f)) showed that aDC were significantly positively correlated with some proinflammatory cytokines, such as CXCL10, C-C motif chemokine ligand 5 (CCL5), and C-C motif chemokine ligand 4 (CCL4) which had been reported to exacerbate IRI during liver transplantation [7, 8]. Taken together, these results indicated that the increase of DCs in aging livers may contribute to the susceptibility of aging livers to IRI.

### 3.5. Screening Genes Simultaneously Correlated to Aging and IRI of Liver Transplantation

**Figure 6:**
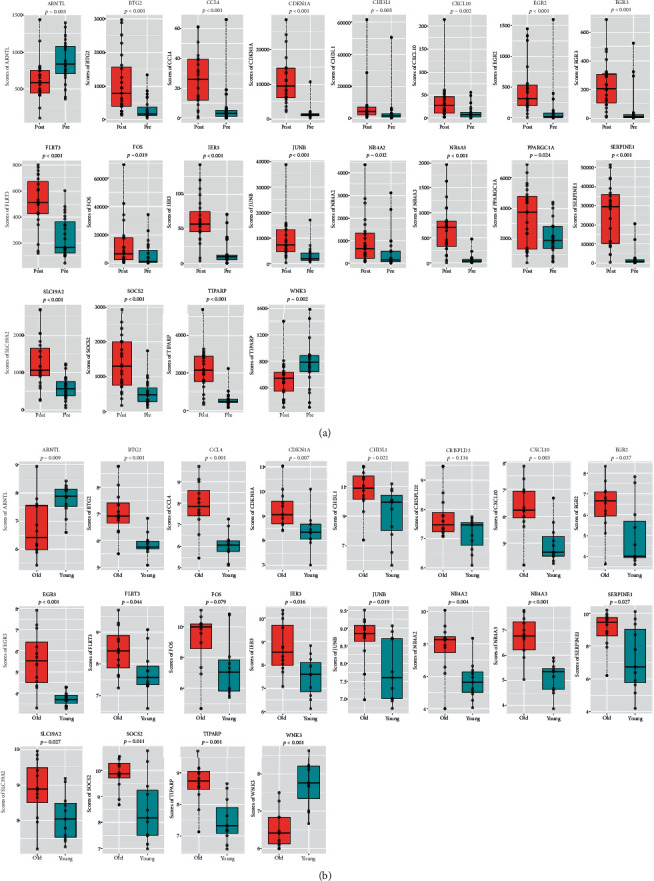
Screening genes simultaneously correlated to IRI and aging livers. (a) The expression profiles of selected genes in liver tissue pre- and posttransplantation. (b) The expression profiles of selected genes in young and aging livers.

Inflammation certainly plays an important role in IRI, but the ability of the tissue itself to cope with external stress also greatly affects the degree of tissue IRI. Compared with young livers, aging livers have an impaired ability to respond to external stress which contributes to the susceptibility of aging livers to IRI. The changes in aging livers are closely related to the insufficient response of aging livers to external stress, while the specific changes, especially in the genetic level, have not yet been fully elucidated. GSE151648, a high-throughput expression profiling dataset of human liver IRI, was used to screen the potential genes correlated to IRI in liver transplantation. Combined with the 65 differential genes obtained in Figure 3(a), we further screened the genes simultaneously correlated to age changes and IRI of liver transplantation. As shown in Figures 6(a) and 6(b), a total of 20 genes had similar expression trends in the livers suffered IRI and aging livers. Among them, ARNTL and WNK lysine deficient protein kinase 1 (WNK) were downregulated in liver tissues suffered IRI and aged liver tissues. Previous study [9] showed that ARNTL can effectively reduce liver steatosis; therefore, we speculated that the decrease of ARNTL in aged livers may aggravate the steatosis which further increased the susceptibility of aging livers to IRI. In other upregulated 18 genes, BTG2 [10] and cyclin-dependent kinase inhibitor 1A (CDKN1A) were associated with proliferation inhibition; CXCL10 [7], CCL4, CHI3L1, IER3, and FOS/JUNB were associated with inflammation; PPARGC1A [11], nuclear receptor subfamily 4 group A member 3 (NR4A3), and suppressor of cytokine signaling 2 (SOCS2) were associated with the regulation of metabolism. Consistent with previous studies [2], aging livers have undergone significant changes in cell proliferation, metabolism, and inflammation compared with young livers, and these changes may be closely related to the aging liver being more susceptible to IRI. Taken together, these results suggested that a series of genes related to cell proliferation, metabolism, and inflammation had similar expression trend in aging livers and livers subjected to IRI, and the changes of these genes may contribute to aging livers being more prone to IRI.

### 3.6. Validation of the Predicted Key Genes with Clinical Samples

**Figure 7:**
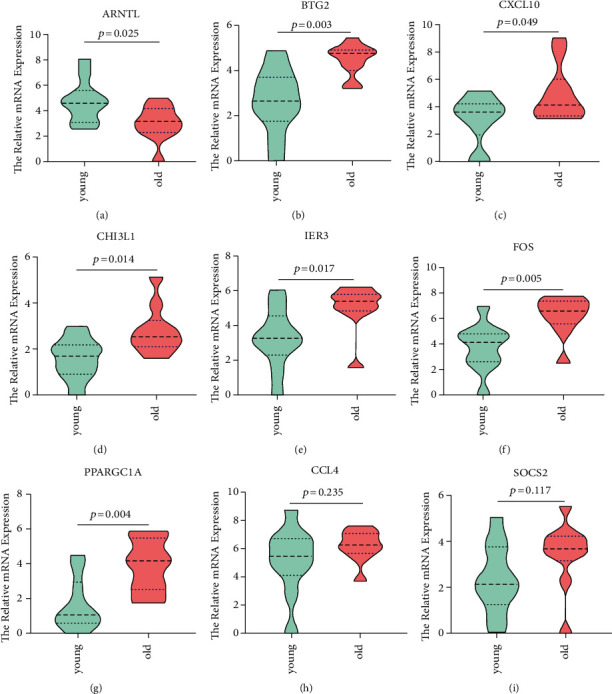
Verification of the expression of selected genes with clinical specimens of our center. qRT-PCR was performed to detect the expression of ARNTL (a), BTG2 (b), CXCL10 (c), CHI3L1 (d), IER3 (e), FOS (f), PPARGC1A (g), CCL4 (h), and SOCS2 (i) in young (*n* = 10) and aging (*n* = 10) livers. *P* < 0.05 was considered statistically significant.

Based on the inherent functions of the above 20 genes (Figure 6), ARNTR, BTG2, CXCL10, CCL4, CHI3L1, IER3, FOS, PPARGC1A, and SOCS2 were selected for further validation. We collected 10 cases of aging liver tissue (donors' age≧50) and 10 cases of young liver tissue (donors' age < 50); then, qRT-PCR was performed to detect the expression of the above-mentioned genes. As shown in Figure 7, ARNTL was significantly downregulated in aging livers, while the expression of BTG2, CXCL10, CHI3L1, IER3, FOS, and PPARGC1A was significantly upregulated in aging livers, which were consistent with the results in Figure 6. There was no significant difference in the expression levels of the other two genes (CCL4 and SOCS2) between young and aging livers. The above results suggested that ARNTL, BTG2, CXCL10, CHI3L1, IER3, FOS, and PPARGC1A may contribute to the susceptibility of aging livers to IRI, which can be used as targets to alleviate IRI in aging livers during transplantation.

### 3.7. Screening of Drugs with Potential to Alleviate IRI in Aging Livers

**Figure 8:**
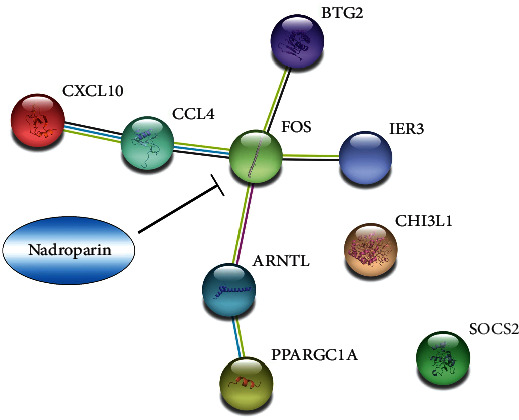
Screening potential drugs to alleviate IRI in aging livers. String map showed that the interaction between 9 selected molecules and FOS was located in the center position. Nadroparin screened in DrugBank was with the potential to inhibit the function of FOS.

DrugBank Online (https://go.drugbank.com/) [12] is a comprehensive, free-to-access, online database containing information on drugs and drug targets. The results of Figures 6 and 7 implied that targeting ARNTL, BTG2, CXCL10, CHI3L1, IER3, FOS, PPARGC1A, CCL4, and SOCS2 may relieve IRI in aging livers. We used DrugBank Online to search for the corresponding drugs for the above molecules one by one and found that nadroparin [13], a low molecular weight heparin, can target to inhibit the function of FOS. What is more interesting was that when we analyzed the interaction between the above-mentioned molecules using STRING (https://string-db.org/), we found that the above-mentioned molecules can form an interaction network except CHI3L1 and SOCS2, and FOS was located in the center position (Figure 8). Nadroparin is usually used for the prophylaxis of thrombotic events and deep vein thrombosis and prevents unstable angina and non-Q-wave myocardial infarction with the characteristics of low price and few adverse effects. This study suggested that nadroparin may also be used to alleviate IRI in aging livers.

## 4. Discussion

With the intensification of aging population worldwide, the proportion of aging livers in the donor pool is increasing. To solve the problem of donor shortage, it is inevitable to accept elderly donor livers in transplantation. However, compared with young livers, the recipients of the livers from old donors showed an increased complications and mortality after transplantation which is closely related to the aging liver being more prone to IRI. With aging, the process of energy metabolism, inflammatory response, and proliferation ability has changed significantly in the livers, which are generally considered to be closely related to the enhanced susceptibility of aging livers to IRI [14, 15]. However, the detail mechanisms underlying this age-mediated hypersensitivity to IRI remain poorly understood. Chun et al.'s research group [16] found that loss of sirtuin 1 and mitofusin 2 contributes to enhanced IRI in aging livers via regulating autophagy. Zhong et al.'s research group [17] reported that aging aggravated liver IRI by promoting hepatocyte necroptosis in an endoplasmic reticulum stress-dependent manner. Li et al.'s research group [18] proved that aging aggravated hepatic IRI by impairing the age-dependent mitophagy function via an insufficient parkin expression. It is urgent to further clarify the potential molecular mechanisms of aging livers being more susceptible to IRI and develop new methods to alleviate IRI, thereby improving the utilization of aging liver in transplantation.

In this study, we firstly integrated four expression profiling datasets (GSE61260, GSE107037, GSE89632, and GSE133815) of human liver tissue with different age composition and found that there were significant differences in the gene expression profiles between young and aging livers. Among them, a series of differential genes were also dysregulated in the livers subjected to IRI, such as ARNTL, BTG2, CXCL10, CHI3L1, IER3, FOS, and PPARGC1A which mainly involved in the regulation of cell proliferation, metabolism, and inflammation. Changes in the expression levels of these genes were further validated in 20 liver tissues collected in our clinical center which suggested that they may be associated with aging livers being more prone to IRI and targeting these genes may alleviate IRI in aging livers.

It is worth mentioning that the above molecules can form an interaction network, and FOS was in the central position. Nadroparin [13] is a low molecular weight heparin used for the prophylaxis of thrombotic events and deep vein thrombosis and prevents unstable angina and non-Q-wave myocardial infarction, which has been used in clinical for many years with the characteristics of low price and few adverse effects. The data in DrugBank Online showed that nadroparin can also target and inhibit the function of FOS which attracted us great attention. The conventional organ preservation solution or perfusion solution contains a certain proportion of anticoagulant, which means that the anticoagulant itself is needed and harmless in organ transplantation. We speculate that adding an appropriate amount of nadroparin to the organ preservation solution or perfusion solution may not only exert an anticoagulant effect but also alleviate the IRI of aging livers by inhibiting the function of FOS, which is very worthy of further research. In addition, Wu et al. [19] proved that acteoside presented protective effects on cerebral IRI through targeting CXCL10. Furuichi et al. [20] found that anti-CXCL10-Ab treat could enhance the proliferation ability of tubular cells in renal after reperfusion. Deng et al. [21] demonstrated that inhibiting the expression of CHI3L1 can strengthen the effect of dexmedetomidine preconditioning to protect against myocardial IRI in mouse models. These studies further suggest that targeting CXCL10 and CHI3L1 may also alleviate IRI in aging livers which deserve further research.

Growing evidences proved that using aged grafts in liver transplantation could lead to a more severe postoperative inflammatory response than the use of young grafts, and the inflammatory response plays a critical role in hepatic IRI [6, 22]. Activation of the Kupffer cells [4, 23] is a central event in the initial phase of IRI, and the activation of neutrophils [24] is in the later phase. The roles of these two cell types in liver IRI had been widely reported, while there was no significant difference in the proportion of them between young and aging livers (supplementary table 1). We wondered whether there were other immune cells associated with aging livers being more susceptible to IRI. In this study, we proved that DC was significantly upregulated in aging livers. A few studies [25, 26] have showed that DC was implicated in the control of IRI and host immune responses following liver transplantation, while the correlation between DC and the susceptibility of aging livers to IRI had not yet been reported. Agrawal et al. [27] reported that tumor necrosis factor alpha (TNF-*α*) and prostaglandins would result in premature DC activation; altering their antigen uptake capacity and TNF-*α* was highly expressed in aging livers. We speculate that combining the increased level of TNF-*α* and upregulated DC numbers in aging livers, the activated DC is significantly increased, thereby triggering inflammatory response and aggravating IRI in aging livers. Appropriate regulation of DC cell function may reduce IRI in aging liver, and it is worth investing more energy in further research.

Our work also has limitations in the screening of differential genes and cell subsets with gene expression microarray data. Due to the batch effects of microarray data that are almost inevitable, it is important to remove batch effects before data is being analyzed. Chen et al. [28] evaluated the effects of 6 batch adjustment methods and proved that ComBat, an empirical Bayes method, had the best effect on removing batch effects. However, due to the data sets downloaded without batch information, we could not perform ComBat.

## 5. Conclusions

In summary, we combined the expression profiling datasets of liver tissues and samples collected in our hospital for the first time to reveal that the changes in the expression of ARNTL, BTG2, CXCL10, CHI3L1, IER3, FOS, and PPARGC1A and the proportion of dendritic cells may be associated with aging livers being more prone to IRI. Nadroparin can be used to mitigate IRI in aging livers by targeting FOS, and regulation of DC activity may also reduce IRI. Our work provided new targets and theoretical basis for developing new methods to alleviate IRI in aging livers.

## Data Availability

The datasets and all experimental materials used in this study are available from the corresponding authors on reasonable request.

## Abbreviations

IRI: Ischemia-reperfusion injury
ARNTL: Aryl hydrocarbon receptor nuclear translocator-like
BTG2: BTG antiproliferation factor 2
CXCL10: C-X-C motif chemokine ligand 10
CHI3L1: Chitinase 3-like 1
IER3: Immediate early response 3
FOS: Fos proto-oncogene, AP-1 transcription factor subunit
PPARGC1A: Peroxisome proliferative activated receptor, gamma, coactivator 1 alpha
DCs: Dendritic cells
USA: The United States of America
HBV: Hepatitis B virus
HCV: Hepatitis C virus
IR: Ischemia-reperfusion
IHC: Immunohistochemistry
EDTA: Ethylenediaminetetraacetic acid
RNA: Ribonucleic acid
qRT-PCR: Quantitative real-time polymerase chain reaction
cDNA: Complementary deoxyribonucleic acid
GAPDH: Glyceraldehyde-3-phosphate dehydrogenase
TP53: Tumor protein p53
aDC: Activated dendritic cells
CCL5: C-C motif chemokine ligand 5
CCL4: C-C motif chemokine ligand 4
WNK: WNK lysine deficient protein kinase 1
CDKN1A: Cyclin-dependent kinase inhibitor 1A
JUNB: JunB proto-oncogene, AP-1 transcription factor subunit
NR4A3: Nuclear receptor subfamily 4 group A member 3
SOCS2: Suppressor of cytokine signaling 2
TNF-*α*: Tumor necrosis factor alpha.

## Supplementary Materials

Supplementary Materials Supplementary Figure 1: the differential gene profile in young and aging livers. The volcano maps were used to exhibit the numbers of differential genes and their related up-/downregulation profiles between young and aging livers of GSE61260 (A), GSE107037 (B), GSE89632 (C), and GSE133815 (D). Supplementary Table 1: the differential genes in young and aging livers. Supplementary Table 2: the composition profiles of 64 common types of stroma and immune cells in young and aging livers. Supplementary Table 3: the list of primers.
