# Supplementary material for: Identify Key Genes Correlated to Ischemia-Reperfusion Injury in Aging Livers
Source: Dis Markers. 2023 Feb 16;2023:4352313. doi: 10.1155/2023/4352313 (PMC9949953; doi:10.1155/2023/4352313)
Supplement: Supplementary Materials — Supplementary Figure 1: the differential gene profile in young and aging livers. The volcano maps were used to exhibit the numbers of differential genes and their related up-/downregulation profiles between young and aging livers of GSE61260 (A), GSE107037 (B), GSE89632 (C), and GSE133815 (D). Supplementary Table 1: the differential genes in young and aging livers. Supplementary Table 2: the composition profiles of 64 common types of stroma and immune cells in young and aging livers. Supplementary Table 3: the list of primers. [file 4352313.f1.pdf]

## Supplementary Fig. 1

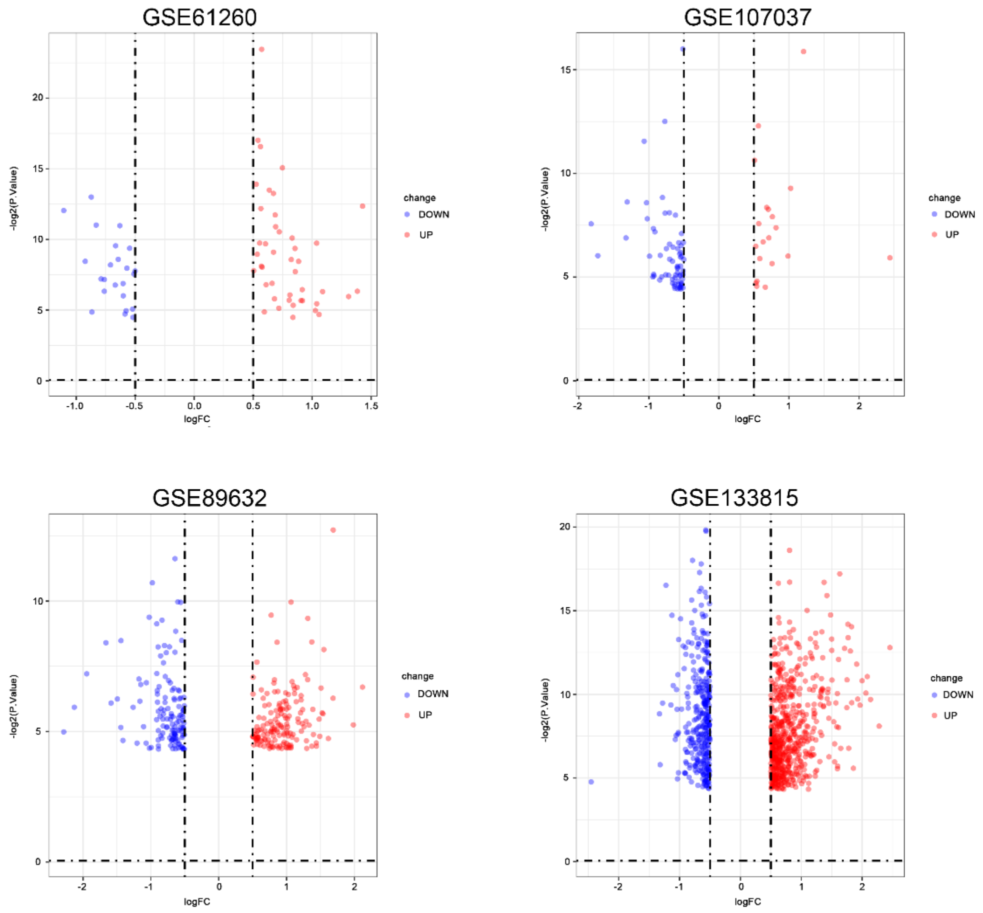


Supplementary Fig. 1. The differential genes profile in young and aging livers. The volcano maps were used to exhibit the numbers of differential genes and their related up/down-regulation profiles between young and aging livers of GSE61260 (A), GSE107037 (B), GSE89632 (C) and GSE133815 (D).

**Supplementary Table.1**

| Supplementary Table.1 the differential genes in young and aging livers | | | |
|---|---|---|---|
| GSE61260 | GSE89632 | GSE133815 | GSE107037 |
| CRP | MT1M | ADH1B | AKR1C2 |
| IGFBP2 | PON2 | HMGCS2 | CPS1 |
| GPX2 | PRDX3 | PLG | ARG1 |
| EGR1 | GSTA2 | ACTB | CHI3L1 |
| FADS2 | NHEDC2 | ADH4 | PLIN2 |
| SERPINE1 | ATP2C1 | A1BG | IGLC1 |
| CDHR2 | IGJ | HSD17B6 | SULT2A1 |
| FADS1 | GCSH | AKR1C2 | MBNL3 |
| IL32 | YPEL2 | CYP3A5 | RHOB |
| OAT | CITED2 | ADH1C | FKBP5 |
| JUNB | SPG21 | IGFBP1 | TSPAN13 |
| SULF2 | BID | SAT1 | ERRFI1 |
| CYP3A43 | UGT2A3 | ADH1A | HGD |
| FOS | SIVA | P4HB | PLPP1 |
| CPS1-IT1 | ENHO | GATM | IRS2 |
| CHI3L1 | SRXN1 | SERPIND1 | ZFAND5 |
| RNU5E-1 | ACOT2 | HSPA5 | LDLR |
| FNDC5 | KHK | CDO1 | ALAS1 |
| SULT1E1 | C19orf60 | PLPP3 | SLCO1B3 |
| FAT1 | SENP2 | FMO3 | IGHM |
| SGCE | SNORD67 | UGP2 | FGGY |
| SPP1 | C1orf122 | LRG1 | TRPM8 |
| IER3 | POLR2D | CFHR4 | IGKC |
| RNU4-2 | RRP15 | UGT2B15 | OAT |
| CXCL10 | UBE2L6 | MCL1 | COBLL1 |
| DDB2 | IFRD2 | ANGPTL3 | N4BP2L1 |
| CDKN1A | LSM12 | PHYH | G6PC |
| KDELR3 | SLC25A30 | PTP4A1 | LGALS4 |
| DDIT4 | MOCS2 | SLC7A2 | SLC25A33 |
| GDF15 | SLC31A1 | TM4SF4 | TEAD1 |
| ZMAT3 | SNORA16A | TRIB1 | RPS4Y1 |
| UBD | AP3M1 | LECT2 | FZD5 |
| SLCO4C1 | FLRT3 | FOLR1 | TSC22D3 |
| GSDMB | TP53 | ACADM | SLC25A15 |
| VIL1 | FASTKD3 | AKR1C3 | PCSK6 |
| HSD17B14 | SMG7 | AQP9 | SOCS2 |
| NCAM2 | SNORA18 | RND3 | PDK4 |
| PEG10 | FOS | GBA3 | TSKU |

| | | | |
|---|---|---|---|
| TSPAN8 | TIMD4 | RNASE4 | SH3BP4 |
| KCNAB2 | SNORD65 | GOT1 | ARHGEF26 |
| TYMS | FAM190B | ADH6 | SYBU |
| CCL3 | STARD3NL | MBNL3 | EFHD1 |
| RND2 | RPS21 | ATF5 | SLC3A1 |
| CD24 | ASPG | HNMT | RORA |
| EGR2 | TRA1P2 | IRS2 | CPEB4 |
| LINC00268 | OAS1 | SSR1 | SLC19A2 |
| KCNB1 | SFMBT1 | RGN | ZBTB16 |
| RPS4Y1 | AGPAT3 | SGK1 | KLF9 |
| NPBWR1 | EARS2 | GHR | SLC1A2 |
| ASCL1 | ENDOG | GADD45B | CRISPLD2 |
| EIF1AY | SMAGP | TSC22D1 | NEDD9 |
| KDM5D | HDLBP | JUND | ARNTL |
| DDX3Y | C7orf20 | ECI2 | TIPARP |
| GABRB3 | PVRL2 | CMPK1 | BACE2 |
| LGI1 | CTBP1 | FKBP5 | PPARGC1A |
| MIR32 | PRIM1 | SRGN | XIST |
| UGT2B17 | CRKRS | GBP1 | CECR2 |
| AKR1B10 | GLE1 | SORL1 | CYP1A1 |
| UTY | MPND | CHI3L1 | CFD |
| SLITRK6 | PPIA | IGLC1 | THBD |
| TXLNGY | SNORA50 | NFE2L2 | ZIC1 |
| CCL4 | ITM2A | CBS | DDX3Y |
| USP9Y | FADS2 | RRBP1 | AGPAT5 |
| SNORA2A | SNORA49 | FMO5 | APOLD1 |
| TAAR3P | FAM195A | KDELR2 | GRHL1 |
| CXCL13 | DDX50 | SDC2 | CYP4F22 |
| | SNORA8 | SLCO1B1 | WNK3 |
| | RPS7 | SPP2 | CCL5 |
| | PSME3 | PPP1CB | EIF1AY |
| | NPLOC4 | CD74 | LURAP1L |
| | C17orf95 | DUSP6 | MYSM1 |
| | LOC645296 | FKBP11 | KDM5D |
| | SNHG9 | ANXA10 | POSTN |
| | BTG2 | PTPN11 | POU2AF1 |
| | SNORD93 | MBNL2 | TSPYL5 |
| | LRRC58 | NARS1 | ISM1 |
| | MXRA7 | ZFP36 | GABRB3 |
| | ATP6V0D1 | LIPA | ZNF518B |
| | SNORA32 | HIPK2 | TXLNGY |
| | SNORA2B | ELL2 | CD2 |
| | SNORD25 | XDH | SLAMF7 |

| | | | |
|---|---|---|---|
| | HHLA3 | ACACB | HKDC1 |
| | C18orf56 | N4BP2L1 | CX3CR1 |
| | TYW1 | THRSP | SLITRK3 |
| | TRUB1 | BHLHE40 | USP9Y |
| | XAB2 | PXMP2 | FREM2 |
| | B3GALTL | C11orf96 | ZFY |
| | CEP290 | DNAJC3 | |
| | SNORA75 | TP53INP1 | |
| | LOC205251 | HLA-DPA1 | |
| | ABCA5 | LIPC | |
| | PHLPP2 | HYOU1 | |
| | C6orf85 | TGFBI | |
| | SNORD117 | CXCL12 | |
| | SNORA72 | KMO | |
| | FAM38B | NFIL3 | |
| | CBLB | STARD10 | |
| | AGXT2L2 | OTC | |
| | SNORD38A | RPL36 | |
| | MIPEP | UGDH | |
| | SNORA41 | SSR3 | |
| | SNORD97 | NR3C1 | |
| | SNORD35A | LPIN2 | |
| | NANP | CCN1 | |
| | GRINL1A | SOCS2 | |
| | SNORA25 | CRELD2 | |
| | PVALB | DLC1 | |
| | MTMR11 | RPN1 | |
| | CLCN4 | PGLYRP2 | |
| | SNORA11 | FST | |
| | FMOD | SEC61A1 | |
| | C6orf142 | ABCA8 | |
| | RGS1 | KLF10 | |
| | SCARNA8 | RASD1 | |
| | SYT7 | HLA-DRA | |
| | SNORD27 | EPHX2 | |
| | LOC729920 | DHRS1 | |
| | C1orf66 | AGXT2 | |
| | IKZF1 | GLS2 | |
| | FAM174B | PSMD14 | |
| | C20orf94 | HTRA1 | |
| | TMEM63A | CCT3 | |
| | C10orf33 | MFAP3L | |
| | BAT4 | VDAC1 | |

| | | | |
|---|---|---|---|
| | SNORA1 | UBA1 | |
| | ODF2L | DGAT2 | |
| | ADAT1 | SLC2A10 | |
| | PIM3 | TSPAN13 | |
| | SNORD1A | TSKU | |
| | GMPPA | IFITM1 | |
| | NUAK2 | IL1RN | |
| | RERG | CTSS | |
| | CDK8 | MS4A6A | |
| | IRAK4 | DNAJB11 | |
| | MADD | EIF1B | |
| | ZNF77 | TENT5A | |
| | POLR2C | TUBB4B | |
| | SCARNA4 | PRKAR2A | |
| | SCARNA16 | SLC6A1 | |
| | SNORA21 | CDKN1A | |
| | CLSTN2 | SRPRB | |
| | SNORD73A | HLF | |
| | MIR644 | PPM1B | |
| | SNORA74A | NUCB2 | |
| | TMEM110 | ABCC2 | |
| | SNORD35B | TM7SF3 | |
| | ARSB | CHD9 | |
| | HOOK2 | C2orf72 | |
| | SNORA14B | EIF3B | |
| | MFN1 | PHLDA1 | |
| | SCARNA3 | ATP5F1D | |
| | SNORA40 | MAFB | |
| | GSTT2 | OIT3 | |
| | SUSD2 | NUCB1 | |
| | C17orf101 | PEMT | |
| | NARFL | UGCG | |
| | RHOJ | CFAP20 | |
| | PTPN13 | PDGFC | |
| | SNORA73A | SLC51A | |
| | WDR89 | DAPK1 | |
| | SNORA9 | RNASET2 | |
| | SLC2A1 | CCT6A | |
| | SNORD10 | TPD52 | |
| | ZNF117 | RNF19A | |
| | C20orf29 | SLC19A2 | |
| | C6orf114 | TCIM | |
| | ITGA8 | GSAP | |

| | | | |
|---|---|---|---|
| | GALNTL2 | MANF | |
| | ACPL2 | ZMAT3 | |
| | CLEC14A | IGKC | |
| | N4BP1 | AMT | |
| | TK1 | NR0B2 | |
| | TRAF3IP1 | MLXIPL | |
| | SIGLEC1 | SND1 | |
| | PODN | GARS1 | |
| | SNORD96A | SLC35B1 | |
| | ADORA2A | TMEM37 | |
| | STC1 | USP22 | |
| | SNORA26 | RETREG1 | |
| | TBCD | TRIM25 | |
| | NTS | CUX2 | |
| | SIGLEC9 | MYC | |
| | SNORD15A | SLIRP | |
| | RIPPLY1 | PNKD | |
| | MAP2 | DCPS | |
| | GUCA2B | SNRK | |
| | DNAJC5 | MYADM | |
| | C15orf63 | FGL2 | |
| | P2RY10 | ABLIM3 | |
| | SNORD6 | FAM110C | |
| | SNORA53 | SMARCA1 | |
| | DNM1 | CKAP4 | |
| | AKR1B10 | ELF1 | |
| | SNORA5C | TKT | |
| | SNORA2A | NME1 | |
| | NR4A2 | PDGFRA | |
| | SNORA27 | C1QC | |
| | CDH11 | CPED1 | |
| | COL15A1 | SLC43A1 | |
| | SCARNA10 | MAP1LC3B | |
| | SC65 | RGS2 | |
| | MAP3K15 | RPL22L1 | |
| | ASAH2 | SFT2D2 | |
| | MAMDC2 | GLUD2 | |
| | COL6A6 | GSN | |
| | RNU5D | XPOT | |
| | SNORA71C | KCNJ8 | |
| | ID4 | HNRNPA0 | |
| | BATF2 | FOS | |
| | GZF1 | RDH10 | |

| | | | |
|---|---|---|---|
| | C5orf56 | JUNB | |
| | SFTPD | C1QB | |
| | SLC16A11 | CRHBP | |
| | MIR30B | YARS1 | |
| | SNORA5A | SOCS3 | |
| | CLIP4 | MS4A7 | |
| | LOC731085 | C1D | |
| | EGR3 | FNDC4 | |
| | GULP1 | TNFAIP3 | |
| | SNORA81 | PUS3 | |
| | SAC | CLYBL | |
| | RTN4RL2 | VMP1 | |
| | SNORA11E | LRRC37A2 | |
| | UFSP1 | EIF4EBP2 | |
| | STON1-GTF2A1L | LYZ | |
| | LOC440944 | TTPAL | |
| | COCH | PTPRC | |
| | MARK1 | RAD23A | |
| | SNORA11B | ZSWIM6 | |
| | FLJ23356 | IER3 | |
| | CTRB2 | SERPINE1 | |
| | NR4A3 | KLF3 | |
| | NOV | GPAT3 | |
| | SNORD22 | NDEL1 | |
| | BCL11B | HARS1 | |
| | SCN7A | CLRN3 | |
| | C1QTNF7 | CREM | |
| | ANGPT1 | TRIM22 | |
| | LOC730413 | FLRT3 | |
| | MIR32 | TIPARP | |
| | MAML3 | SETD5 | |
| | C17orf103 | BMS1P1 | |
| | TFEB | IRAK1 | |
| | TAGAP | CSTA | |
| | SNORA11D | P4HA1 | |
| | SNORD38B | DHCR7 | |
| | KIF4A | GFPT1 | |
| | KCNA5 | COASY | |
| | PLP1 | IVNS1ABP | |
| | CHRM3 | FAM98A | |
| | SNORD92 | ATP1B3 | |
| | SNORA52 | TSTA3 | |
| | P2RX3 | UGGT1 | |

| | | | |
|---|---|---|---|
| | SNORA38 | MASP1 | |
| | MYO1G | SIK2 | |
| | KRT18 | SDF2L1 | |
| | L1TD1 | SLC23A2 | |
| | SNORD9 | SLC33A1 | |
| | LOC389607 | JCHAIN | |
| | KAZALD1 | CITED2 | |
| | MRVI1 | REL | |
| | SNORA58 | ACSM3 | |
| | MUC20 | ARRDC4 | |
| | MMP16 | ZC3HAV1 | |
| | PORCN | HLA-DPB1 | |
| | FMN1 | INSIG2 | |
| | SNORD98 | IKBKG | |
| | RPESP | LUM | |
| | NDUFB1 | GRAMD4 | |
| | CRTC1 | GPAT4 | |
| | SLC45A2 | DESI1 | |
| | PLD5 | GADD45G | |
| | BOC | RBM7 | |
| | LSAMP | PPARGC1A | |
| | TEKT3 | GREM2 | |
| | KIAA1683 | LGALS4 | |
| | DTX3 | COPE | |
| | INSC | MANEA | |
| | FOLR3 | CHMP1B | |
| | TAS2R13 | ESR1 | |
| | LOC728448 | FRMD4B | |
| | SEMA3G | CD55 | |
| | SOX10 | PTOV1 | |
| | FLJ35024 | MARCKS | |
| | ADAM23 | LURAP1L | |
| | SCARNA2 | CSRNP1 | |
| | HMHB1 | GPT2 | |
| | OR51F1 | SHB | |
| | TAC1 | GSTA3 | |
| | PLN | ARFIP2 | |
| | ADAM33 | LYVE1 | |
| | IL2 | GAREM1 | |
| | | TRIM15 | |
| | | CLTRN | |
| | | VCAM1 | |
| | | MOCS2 | |

| | | | |
|---|---|---|---|
| | | LOC728613 | |
| | | CD4 | |
| | | CLOCK | |
| | | RND1 | |
| | | ATF3 | |
| | | KLF4 | |
| | | SRXN1 | |
| | | IFRD1 | |
| | | MAPRE1 | |
| | | FETUB | |
| | | FKBP4 | |
| | | GRINA | |
| | | ENPP2 | |
| | | HM13 | |
| | | RHPN2 | |
| | | FRY | |
| | | CCDC3 | |
| | | MTMR4 | |
| | | RGS1 | |
| | | HSPH1 | |
| | | PCOLCE | |
| | | HES1 | |
| | | AKAP12 | |
| | | NR1D2 | |
| | | MAFK | |
| | | CSF1R | |
| | | MARCO | |
| | | VNN3 | |
| | | CD53 | |
| | | KEAP1 | |
| | | PROZ | |
| | | SKAP2 | |
| | | KIAA0100 | |
| | | PLCG2 | |
| | | PFKFB3 | |
| | | PER2 | |
| | | IFI16 | |
| | | RTKN | |
| | | AKT1 | |
| | | SFXN4 | |
| | | SRD5A2 | |
| | | DDX39A | |
| | | ALDH1B1 | |

| | | | |
|---|---|---|---|
| | | ESRRA | |
| | | SEC24C | |
| | | KIAA0930 | |
| | | BASP1 | |
| | | RPRD2 | |
| | | THBS1 | |
| | | INHBB | |
| | | CCN2 | |
| | | ENC1 | |
| | | CCNDBP1 | |
| | | SEMA4G | |
| | | CRISPLD2 | |
| | | PTPRM | |
| | | ICAM1 | |
| | | MATN2 | |
| | | ZBED3 | |
| | | CCDC117 | |
| | | NEDD9 | |
| | | C3orf85 | |
| | | SERPINB9 | |
| | | SGMS2 | |
| | | ECM2 | |
| | | CLEC2B | |
| | | DEXI | |
| | | SOCS6 | |
| | | COG3 | |
| | | TYROBP | |
| | | ATF1 | |
| | | CXCR4 | |
| | | CXCL16 | |
| | | EVL | |
| | | AK1 | |
| | | NT5E | |
| | | JMY | |
| | | NANS | |
| | | CCL2 | |
| | | LPIN1 | |
| | | PPP1R14B | |
| | | RNF144B | |
| | | ILRUN | |
| | | FBXO3 | |
| | | IRF8 | |
| | | GSR | |

| | | | |
|---|---|---|---|
| | | BNIP2 | |
| | | RGS3 | |
| | | AVPI1 | |
| | | HLA-DMB | |
| | | EMP1 | |
| | | NREP | |
| | | SIRT5 | |
| | | TM7SF2 | |
| | | IGFBP5 | |
| | | GOLGA3 | |
| | | ARNTL | |
| | | ARID5B | |
| | | OGFOD1 | |
| | | MPEG1 | |
| | | PAK2 | |
| | | TMCO3 | |
| | | ZBTB10 | |
| | | NR4A1 | |
| | | CD36 | |
| | | RELL1 | |
| | | C9orf72 | |
| | | ZNF331 | |
| | | FAM76A | |
| | | ANXA1 | |
| | | INHBA | |
| | | MAVS | |
| | | DMXL2 | |
| | | TNS2 | |
| | | STIP1 | |
| | | ELF3 | |
| | | CFP | |
| | | MAP2K2 | |
| | | GPNMB | |
| | | FAS | |
| | | SLC3A1 | |
| | | CDA | |
| | | ABCG2 | |
| | | FOSB | |
| | | WNK3 | |
| | | CMTM4 | |
| | | RALB | |
| | | ANKRD37 | |
| | | AMFR | |

| | | | |
|---|---|---|---|
| | | CALHM6 | |
| | | BAIAP2L1 | |
| | | SPRY2 | |
| | | STARD5 | |
| | | PMM2 | |
| | | PGM2 | |
| | | PLAC8 | |
| | | PALM3 | |
| | | SERTAD1 | |
| | | FPR1 | |
| | | ITGB2 | |
| | | HLA-DQB1 | |
| | | APOL2 | |
| | | CCL4 | |
| | | MCM3AP | |
| | | CCDC69 | |
| | | CASP4 | |
| | | HLA-DMA | |
| | | ZG16 | |
| | | SLC22A9 | |
| | | POLRMT | |
| | | CDK2AP2 | |
| | | SEPHS1 | |
| | | C3AR1 | |
| | | LOC102723493 | |
| | | HOMER2 | |
| | | FCGR2C | |
| | | CD44 | |
| | | GPR153 | |
| | | CNGA1 | |
| | | CD5L | |
| | | VIPR1 | |
| | | NACC2 | |
| | | PLEK | |
| | | MEF2C | |
| | | GPCPD1 | |
| | | ARNT | |
| | | BRCC3 | |
| | | SRM | |
| | | GEM | |
| | | DOCK7 | |
| | | SIRT1 | |
| | | SH2B3 | |

| | | | |
|---|---|---|---|
| | | DTWD1 | |
| | | TUBB6 | |
| | | THNSL2 | |
| | | DBH-AS1 | |
| | | PSD4 | |
| | | RPH3AL | |
| | | TLR2 | |
| | | ACSM2A | |
| | | DUSP5 | |
| | | MAZ | |
| | | CD300A | |
| | | VWA1 | |
| | | ERBB2 | |
| | | CXCL8 | |
| | | SAMD5 | |
| | | SGMS1 | |
| | | NUTF2 | |
| | | NR4A2 | |
| | | CCR1 | |
| | | TENT5C | |
| | | UNC119B | |
| | | EMILIN3 | |
| | | RAB33B | |
| | | FEZ1 | |
| | | ADA2 | |
| | | PLD1 | |
| | | FAM102A | |
| | | FOSL2 | |
| | | TP53BP2 | |
| | | DLEU2 | |
| | | COPG1 | |
| | | SMARCD2 | |
| | | SNRPA | |
| | | ABCB6 | |
| | | STK38L | |
| | | FCER1G | |
| | | EPHA2 | |
| | | MAFF | |
| | | ASCC2 | |
| | | TRIM13 | |
| | | PRUNE1 | |
| | | MOGAT1 | |
| | | ADGRE5 | |

| | | | |
|---|---|---|---|
| | | NEURL1B | |
| | | TCEA2 | |
| | | RGCC | |
| | | CASP1 | |
| | | LOC101928505 | |
| | | ADRB2 | |
| | | FOLR2 | |
| | | PUS7 | |
| | | DHRS13 | |
| | | TNFSF13B | |
| | | GPR137B | |
| | | SDC3 | |
| | | VCAN | |
| | | ARL4C | |
| | | CCDC85C | |
| | | FTSJ1 | |
| | | SULT1B1 | |
| | | AXL | |
| | | RIOX1 | |
| | | IER5 | |
| | | GNB4 | |
| | | PRR34-AS1 | |
| | | CTSK | |
| | | STK17B | |
| | | FANCC | |
| | | C5AR1 | |
| | | SVBP | |
| | | CPVL | |
| | | PPP1R15A | |
| | | IL33 | |
| | | COLEC10 | |
| | | CD84 | |
| | | MRPL2 | |
| | | SLC2A9 | |
| | | SH3RF2 | |
| | | PAK1IP1 | |
| | | STAB2 | |
| | | ADSS1 | |
| | | GASK1B | |
| | | CCL5 | |
| | | FILIP1L | |
| | | SLC4A2 | |
| | | TSC2 | |

| | | | |
|---|---|---|---|
| | | PTTG1 | |
| | | BTG2 | |
| | | CD1D | |
| | | ZMYM6 | |
| | | SEL1L3 | |
| | | ADAMTS17 | |
| | | DDB2 | |
| | | LINC02499 | |
| | | FEN1 | |
| | | PLAUR | |
| | | RLF | |
| | | AKT1S1 | |
| | | GJA1 | |
| | | COMTD1 | |
| | | TFPI2 | |
| | | EMC1 | |
| | | SLC9A9 | |
| | | TAPBPL | |
| | | CLEC7A | |
| | | DDX60L | |
| | | WEE1 | |
| | | C12orf49 | |
| | | ZCCHC10 | |
| | | FIGN | |
| | | HOTS | |
| | | FICD | |
| | | LEPR | |
| | | SUCNR1 | |
| | | OSGIN2 | |
| | | TOMM40 | |
| | | HNF4G | |
| | | FAM83H | |
| | | TTL | |
| | | IL10RA | |
| | | CETP | |
| | | DCTN5 | |
| | | TSLP | |
| | | TXLNA | |
| | | MAMDC4 | |
| | | KBTBD7 | |
| | | GLCE | |
| | | ABHD5 | |
| | | ZNF587B | |

| | | | |
|---|---|---|---|
| | | TLN1 | |
| | | DDX60 | |
| | | SEPTIN6 | |
| | | TOMM40L | |
| | | HSPA4L | |
| | | ANKRD44 | |
| | | PDE4B | |
| | | LINC-PINT | |
| | | KCNK5 | |
| | | DOK4 | |
| | | BACE2 | |
| | | LCP2 | |
| | | NINJ2 | |
| | | MED30 | |
| | | DKK3 | |
| | | C8orf33 | |
| | | NAPSB | |
| | | FYB1 | |
| | | C1orf226 | |
| | | SLC27A4 | |
| | | SOX4 | |
| | | ABCG1 | |
| | | SPATA6L | |
| | | NOS1AP | |
| | | FANCF | |
| | | NRG1 | |
| | | GGTA1P | |
| | | C2orf68 | |
| | | METRNL | |
| | | HCK | |
| | | TMEM273 | |
| | | GBF1 | |
| | | BCR | |
| | | TBX15 | |
| | | MMP19 | |
| | | PIGW | |
| | | ZMYND19 | |
| | | TLR4 | |
| | | NCLN | |
| | | LY86 | |
| | | THEMIS2 | |
| | | LOC100505985 | |
| | | NME7 | |

| | | | |
|---|---|---|---|
| | | TNFAIP8 | |
| | | SLC19A3 | |
| | | CFD | |
| | | DNALI1 | |
| | | CENPBD1P1 | |
| | | MYOF | |
| | | PDLIM3 | |
| | | FAM102B | |
| | | WDR5 | |
| | | SYPL2 | |
| | | KLHDC7A | |
| | | SOCS7 | |
| | | SOX9 | |
| | | ASAP3 | |
| | | NR4A3 | |
| | | RPS6KA2 | |
| | | FOXQ1 | |
| | | HLA-DQA1 | |
| | | TLE4 | |
| | | ALOX5AP | |
| | | IYD | |
| | | SLC2A3 | |
| | | SAMSN1 | |
| | | MICOS10P1 | |
| | | ZNF746 | |
| | | PLA2G4C | |
| | | CDH5 | |
| | | LOC102606465 | |
| | | DNMT3L | |
| | | EPB41L4A | |
| | | NUSAP1 | |
| | | CXCL1 | |
| | | TLR3 | |
| | | NPAS2 | |
| | | MSTO1 | |
| | | STAT4 | |
| | | PLAGL1 | |
| | | HEY2 | |
| | | BCAT2 | |
| | | IL13RA2 | |
| | | LAMA2 | |
| | | WFS1 | |
| | | SCAPER | |

| | | | |
|---|---|---|---|
| | | RGS12 | |
| | | PPP1R3E | |
| | | PMS2P5 | |
| | | EMCN | |
| | | CYTIP | |
| | | IGSF6 | |
| | | SOX7 | |
| | | ESPL1 | |
| | | SLC36A4 | |
| | | EME1 | |
| | | GLIDR | |
| | | LMBR1 | |
| | | CARD16 | |
| | | EPSTI1 | |
| | | PRR18 | |
| | | SAC3D1 | |
| | | HBEGF | |
| | | PTGER4 | |
| | | CEP78 | |
| | | ASB9 | |
| | | KCNB1 | |
| | | IGHD | |
| | | ECE2 | |
| | | ZC2HC1C | |
| | | ANGPTL2 | |
| | | EGR2 | |
| | | RIPOR3 | |
| | | DUSP2 | |
| | | CD83 | |
| | | CXCL10 | |
| | | FCGR3B | |
| | | ANXA13 | |
| | | PHF12 | |
| | | NCF2 | |
| | | TBC1D16 | |
| | | GZMK | |
| | | PLK3 | |
| | | MAP3K8 | |
| | | PLA2G7 | |
| | | TNFRSF11B | |
| | | PTPRN2 | |
| | | LINC00888 | |
| | | PLEKHH1 | |

| | | | |
|---|---|---|---|
| | | CLIC2 | |
| | | FITM1 | |
| | | ICAM2 | |
| | | ZNF514 | |
| | | MARCHF1 | |
| | | TBXAS1 | |
| | | SPDYE2 | |
| | | LRR1 | |
| | | LIF | |
| | | KBTBD6 | |
| | | PLA2G5 | |
| | | PTPRE | |
| | | GPR183 | |
| | | P2RX7 | |
| | | IGLV1-44 | |
| | | IL1B | |
| | | LILRB5 | |
| | | ITPKB | |
| | | CCDC137 | |
| | | IPO4 | |
| | | FPR3 | |
| | | CD86 | |
| | | MPV17L2 | |
| | | FAM49A | |
| | | TRIM10 | |
| | | LRRK2 | |
| | | DIRAS3 | |
| | | HOMER1 | |
| | | MAD1L1 | |
| | | MAP2 | |
| | | CCDC146 | |
| | | OAS2 | |
| | | MOGAT3 | |
| | | PDE9A | |
| | | IGLJ3 | |
| | | CR1 | |
| | | CYBB | |
| | | MSR1 | |
| | | SLC4A7 | |
| | | MX2 | |
| | | PPM1L | |
| | | PPM1D | |
| | | HAND2-AS1 | |

| | | | |
|---|---|---|---|
| | | GZMA | |
| | | CAMSAP3 | |
| | | CHEK2 | |
| | | SPRED1 | |
| | | GABRB3 | |
| | | NOCT | |
| | | C4orf33 | |
| | | P2RY13 | |
| | | DNLZ | |
| | | UBXN6 | |
| | | SLC16A4 | |
| | | SULF1 | |
| | | NABP2 | |
| | | MNDA | |
| | | FCAMR | |
| | | VNN2 | |
| | | BEX1 | |
| | | VMO1 | |
| | | TP53 | |
| | | HSPB8 | |
| | | CNOT3 | |
| | | CLIP4 | |
| | | SLC2A12 | |
| | | MFAP4 | |
| | | SLC25A34 | |
| | | KLHL13 | |
| | | PHLDA2 | |
| | | CXCL13 | |
| | | TIMD4 | |
| | | SLC16A14 | |
| | | ZMAT1 | |
| | | CD72 | |
| | | KLF5 | |
| | | GBP5 | |
| | | RAB27B | |
| | | SPATA18 | |
| | | TJP3 | |
| | | ZNF518B | |
| | | SELL | |
| | | SGO2 | |
| | | TNFSF11 | |
| | | EPB41L3 | |
| | | CCL18 | |

| | | | |
|---|---|---|---|
| | | FBLN7 | |
| | | TMEM178A | |
| | | ZNF267 | |
| | | ADGRE2 | |
| | | CPA3 | |
| | | MATR3 | |
| | | FCN1 | |
| | | FCGR1B | |
| | | IGHG1 | |
| | | PLAT | |
| | | SLC35G2 | |
| | | DSG1 | |
| | | IFI44L | |
| | | GPRIN3 | |
| | | GPR160 | |
| | | FAM83D | |
| | | IL10 | |
| | | LRIG3 | |
| | | AADACP1 | |
| | | TTLL7 | |
| | | GASK1A | |
| | | IL6 | |
| | | LY9 | |
| | | SLFN11 | |
| | | LOC401261 | |
| | | EGR3 | |
| | | FILIP1 | |
| | | LOC100505874 | |
| | | SLC44A3 | |
| | | CSF2RB | |
| | | MTSS2 | |
| | | BABAM2-AS1 | |
| | | IFT81 | |
| | | IL18 | |
| | | PTGS2 | |
| | | PSPH | |
| | | PIK3CG | |
| | | NOP14-AS1 | |
| | | ZNF605 | |
| | | RACGAP1 | |
| | | MCTP1 | |
| | | ANKRD29 | |
| | | SCN9A | |

| | | | |
|---|---|---|---|
| | | EPHA3 | |
| | | ITK | |
| | | TAPT1-AS1 | |
| | | TOX2 | |
| | | FCGR2A | |
| | | SLC16A6 | |
| | | ADGRG2 | |
| | | PMAIP1 | |
| | | HDAC9 | |
| | | EZH2 | |
| | | MACROD2 | |
| | | RRAD | |
| | | ADGRE1 | |
| | | TOP2A | |
| | | TLR1 | |
| | | CD69 | |
| | | CH25H | |
| | | LOC101928837 | |
| | | CXCL3 | |
| | | ANKRD36 | |
| | | RSAD2 | |
| | | HCAR3 | |
| | | ST8SIA4 | |
| | | FREM2 | |
| | | PTGER2 | |
| | | LGSN | |
| | | PRC1 | |
| | | SCN7A | |
| | | CENPU | |
| | | MCM8 | |
| | | LHX2 | |
| | | BCL2A1 | |
| | | STX11 | |
| | | RASGRP1 | |
| | | HTR2B | |
| | | PSRC1 | |
| | | KRT23 | |
| | | RUBCNL | |
| | | CCNB1 | |
| | | HMMR | |
| | | FNTB | |
| | | LOC100287497 | |
| | | CYSLTR1 | |

| | | | |
|---|---|---|---|
| | | BTBD16 | |
| | | HAS2 | |
| | | APCDD1 | |
| | | PIP4P2 | |
| | | LY75 | |
| | | CCL8 | |
| | | TREH | |
| | | AREG | |
| | | SLC18A2 | |
| | | TMEM71 | |
| | | MELK | |
| | | ADRB1 | |
| | | CCNA2 | |
| | | MLF1 | |
| | | PINK1-AS | |
| | | BAALC | |
| | | AGBL2 | |
| | | CDC20 | |
| | | TNFAIP6 | |
| | | ASPM | |
| | | ESRRG | |
| | | BHLHE41 | |
| | | SOX17 | |
| | | NDST3 | |
| | | CPLX1 | |
| | | HJURP | |
| | | MIR100HG | |
| | | MAMDC2 | |
| | | ARL14 | |
| | | C16orf54 | |
| | | NDC80 | |
| | | QPCT | |
| | | CDC6 | |
| | | DNAJC3-DT | |
| | | GINS1 | |
| | | SDCBPP2 | |
| | | THAP2 | |
| | | GREM1 | |
| | | NLRP3 | |
| | | NCAPG | |
| | | KIF20A | |
| | | FCER1A | |
| | | LOC100272217 | |

| | | | |
|---|---|---|---|
| | | ITGA4 | |
| | | TTK | |
| | | NEK2 | |
| | | CENPK | |
| | | CLEC5A | |
| | | OLR1 | |

| Supplementary Table.2 the composition profiles of 64 common types of stroma and immune cells in young and aging livers | | | | | | | | | | | | |
|---|---|---|---|---|---|---|---|---|---|---|---|---|
| | GSE61260 | | | GSE89632 | | | GSE107037 | | | GSE133815 | | |
| | Old (n=33) | Young (n=33) | p | Old (n=2) | Young (n=22) | p | Old (n=22) | Young (n=11) | p | Old (n=12) | Young (n=11) | p |
| aDC | 0.07 (0.03) | 0.05 (0.04) | 0.04 | 0.06 (0.00) | 0.08 (0.03) | 0.447 | 0.07 (0.05) | 0.06 (0.05) | 0.531 | 0.09 (0.03) | 0.03 (0.03) | <0.001 |
| Adipocytes | 0.07 (0.02) | 0.08 (0.02) | 0.79 | 0.01 (0.01) | 0.01 (0.01) | 0.47 | 0.01 (0.01) | 0.02 (0.02) | 0.065 | 0.07 (0.03) | 0.08 (0.03) | 0.489 |
| Astrocytes | 0.00 (0.00) | 0.00 (0.00) | 0.42 | 0.07 (0.06) | 0.04 (0.04) | 0.206 | 0.00 (0.00) | 0.00 (0.00) | 0.017 | 0.01 (0.01) | 0.00 (0.00) | 0.176 |
| B-cells | 0.04 (0.04) | 0.03 (0.02) | 0.64 | 0.00 (0.00) | 0.00 (0.01) | 0.629 | 0.02 (0.02) | 0.01 (0.01) | 0.184 | 0.01 (0.01) | 0.01 (0.01) | 0.754 |
| Basophils | 0.05 (0.05) | 0.06 (0.04) | 0.79 | 0.17 (0.01) | 0.23 (0.15) | 0.609 | 0.07 (0.11) | 0.03 (0.05) | 0.257 | 0.05 (0.04) | 0.03 (0.04) | 0.29 |
| CD4+ memory | 0.13 (0.07) | 0.13 (0.06) | 0.62 | 0.10 (0.05) | 0.05 (0.05) | 0.12 | 0.16 (0.09) | 0.17 (0.06) | 0.758 | 0.06 (0.04) | 0.03 (0.03) | 0.043 |
| CD4+ naïve T-cells | 0.01 (0.02) | 0.01 (0.02) | 0.99 | 0.00 (0.00) | 0.00 (0.00) | 0.679 | 0.02 (0.03) | 0.01 (0.01) | 0.119 | 0.00 (0.01) | 0.01 (0.01) | 0.101 |
| CD4+ T-cells | 0.04 (0.05) | 0.04 (0.03) | 0.79 | 0.08 (0.03) | 0.04 (0.03) | 0.078 | 0.08 (0.05) | 0.08 (0.04) | 0.784 | 0.03 (0.02) | 0.03 (0.03) | 0.675 |
| CD4+ Tcm | 0.01 (0.01) | 0.01 (0.02) | 0.23 | 0.00 (0.00) | 0.01 (0.02) | 0.757 | 0.03 (0.04) | 0.02 (0.03) | 0.499 | 0.00 (0.01) | 0.01 (0.02) | 0.402 |
| CD4+ Tem | 0.03 (0.03) | 0.02 (0.02) | 0.26 | 0.08 (0.06) | 0.05 (0.04) | 0.411 | 0.04 (0.04) | 0.03 (0.03) | 0.13 | 0.05 (0.03) | 0.03 (0.03) | 0.158 |
| CD8+ naive T- | 0.06 (0.03) | 0.07 (0.03) | 0.04 | 0.07 (0.05) | 0.09 (0.03) | 0.566 | 0.04 (0.03) | 0.04 (0.03) | 0.836 | 0.01 (0.02) | 0.02 (0.03) | 0.264 |
| CD8+ T-cells | 0.03 (0.04) | 0.04 (0.04) | 0.58 | 0.08 (0.04) | 0.07 (0.06) | 0.863 | 0.01 (0.01) | 0.01 (0.01) | 0.296 | 0.03 (0.03) | 0.01 (0.02) | 0.043 |
| CD8+ Tcm | 0.02 (0.04) | 0.01 (0.02) | 0.13 | 0.04 (0.06) | 0.01 (0.02) | 0.132 | 0.00 (0.01) | 0.00 (0.00) | 0.397 | 0.05 (0.03) | 0.01 (0.01) | 0.002 |
| CD8+ Tem | 0.04 (0.03) | 0.03 (0.04) | 0.34 | 0.05 (0.06) | 0.06 (0.05) | 0.774 | 0.02 (0.05) | 0.00 (0.00) | 0.123 | 0.02 (0.02) | 0.01 (0.02) | 0.114 |
| cDC | 0.02 (0.03) | 0.03 (0.03) | 0.65 | 0.02 (0.03) | 0.00 (0.01) | 0.095 | 0.03 (0.02) | 0.01 (0.02) | 0.135 | 0.00 (0.01) | 0.00 (0.00) | 0.15 |
| Chondrocytes | 0.01 (0.01) | 0.01 (0.01) | 0.31 | 0.09 (0.02) | 0.08 (0.05) | 0.624 | 0.00 (0.01) | 0.00 (0.00) | 0.488 | 0.01 (0.01) | 0.01 (0.02) | 0.491 |
| Class-switched memory B-cells | 0.02 (0.03) | 0.03 (0.02) | 0.9 | 0.01 (0.00) | 0.01 (0.02) | 0.568 | 0.02 (0.03) | 0.01 (0.01) | 0.196 | 0.01 (0.01) | 0.00 (0.01) | 0.267 |
| CLP | 0.09 (0.03) | 0.09 (0.03) | 0.34 | 0.06 (0.03) | 0.06 (0.03) | 0.912 | 0.19 (0.07) | 0.21 (0.04) | 0.324 | 0.06 (0.03) | 0.06 (0.04) | 0.866 |
| CMP | 0.01 (0.01) | 0.01 (0.01) | 0.28 | 0.02 (0.03) | 0.03 (0.05) | 0.807 | 0.01 (0.04) | 0.01 (0.01) | 0.529 | 0.01 (0.01) | 0.01 (0.01) | 0.473 |
| DC | 0.04 (0.03) | 0.03 (0.03) | 0.2 | 0.06 (0.01) | 0.06 (0.02) | 0.849 | 0.04 (0.04) | 0.02 (0.02) | 0.161 | 0.05 (0.02) | 0.01 (0.01) | <0.001 |
| Endothelial cell | 0.05 (0.03) | 0.07 (0.05) | 0.11 | 0.08 (0.06) | 0.04 (0.03) | 0.066 | 0.09 (0.06) | 0.09 (0.05) | 0.953 | 0.05 (0.05) | 0.05 (0.02) | 0.988 |
| Eosinophils | 0.11 (0.06) | 0.11 (0.06) | 0.97 | 0.12 (0.05) | 0.13 (0.07) | 0.921 | 0.10 (0.17) | 0.06 (0.05) | 0.497 | 0.08 (0.04) | 0.07 (0.04) | 0.508 |
| Epithelial cells | 0.07 (0.04) | 0.05 (0.03) | 0.06 | 0.07 (0.03) | 0.08 (0.03) | 0.742 | 0.07 (0.05) | 0.04 (0.04) | 0.16 | 0.06 (0.03) | 0.05 (0.03) | 0.832 |
| Erythrocytes | 0.00 (0.01) | 0.00 (0.01) | 0.91 | 0.01 (0.02) | 0.01 (0.02) | 0.984 | 0.00 (0.02) | 0.00 (0.01) | 0.829 | 0.00 (0.00) | 0.02 (0.02) | 0.014 |
| Fibroblasts | 0.06 (0.05) | 0.05 (0.06) | 0.8 | 0.06 (0.04) | 0.04 (0.06) | 0.503 | 0.01 (0.04) | 0.00 (0.00) | 0.488 | 0.02 (0.03) | 0.00 (0.01) | 0.11 |
| GMP | 0.06 (0.03) | 0.05 (0.03) | 0.18 | 0.03 (0.04) | 0.03 (0.03) | 0.829 | 0.03 (0.03) | 0.05 (0.04) | 0.122 | 0.01 (0.02) | 0.01 (0.01) | 0.358 |
| Hepatocytes | 0.03 (0.01) | 0.03 (0.01) | 0.84 | 0.08 (0.01) | 0.08 (0.03) | 0.79 | 0.09 (0.04) | 0.11 (0.02) | 0.202 | 0.02 (0.02) | 0.02 (0.01) | 0.269 |
| HSC | 0.19 (0.06) | 0.20 (0.07) | 0.62 | 0.10 (0.08) | 0.12 (0.05) | 0.719 | 0.16 (0.16) | 0.21 (0.11) | 0.366 | 0.14 (0.07) | 0.08 (0.06) | 0.046 |
| iDC | 0.03 (0.02) | 0.02 (0.03) | 0.45 | 0.04 (0.02) | 0.05 (0.04) | 0.679 | 0.05 (0.05) | 0.03 (0.02) | 0.414 | 0.03 (0.02) | 0.02 (0.02) | 0.504 |
| Keratinocytes | 0.03 (0.02) | 0.02 (0.02) | 0.1 | 0.03 (0.02) | 0.04 (0.04) | 0.673 | 0.04 (0.04) | 0.03 (0.02) | 0.296 | 0.03 (0.01) | 0.02 (0.01) | 0.199 |
| ly Endothelial c | 0.05 (0.03) | 0.07 (0.03) | 0.01 | 0.08 (0.07) | 0.06 (0.04) | 0.489 | 0.07 (0.06) | 0.05 (0.04) | 0.5 | 0.04 (0.04) | 0.05 (0.03) | 0.753 |
| Macrophages | 0.08 (0.05) | 0.06 (0.04) | 0.07 | 0.01 (0.02) | 0.03 (0.02) | 0.35 | 0.08 (0.05) | 0.08 (0.05) | 0.945 | 0.07 (0.04) | 0.06 (0.05) | 0.473 |
| Macrophages M1 | 0.02 (0.01) | 0.02 (0.01) | 0.16 | 0.01 (0.01) | 0.01 (0.01) | 0.951 | 0.02 (0.01) | 0.02 (0.01) | 0.632 | 0.02 (0.01) | 0.01 (0.01) | 0.028 |
| Macrophages M2 | 0.09 (0.06) | 0.06 (0.03) | 0.04 | 0.02 (0.02) | 0.04 (0.03) | 0.399 | 0.05 (0.04) | 0.03 (0.04) | 0.244 | 0.06 (0.04) | 0.07 (0.07) | 0.741 |
| Mast cells | 0.01 (0.01) | 0.01 (0.01) | 0.59 | 0.01 (0.01) | 0.01 (0.01) | 0.577 | 0.01 (0.01) | 0.00 (0.01) | 0.256 | 0.01 (0.00) | 0.01 (0.01) | 0.035 |
| Megakaryocyte | 0.04 (0.02) | 0.05 (0.02) | 0.87 | 0.04 (0.03) | 0.05 (0.04) | 0.588 | 0.13 (0.06) | 0.13 (0.05) | 0.98 | 0.07 (0.04) | 0.03 (0.02) | 0.009 |
| Melanocytes | 0.02 (0.01) | 0.02 (0.01) | 0.88 | 0.03 (0.02) | 0.01 (0.02) | 0.078 | 0.02 (0.03) | 0.01 (0.01) | 0.241 | 0.02 (0.01) | 0.02 (0.01) | 0.653 |
| Memory B-cell | 0.00 (0.01) | 0.00 (0.00) | 0.11 | 0.00 (0.00) | 0.00 (0.00) | 0.516 | 0.01 (0.02) | 0.01 (0.01) | 0.218 | 0.00 (0.01) | 0.00 (0.01) | 0.924 |
| MEP | 0.04 (0.03) | 0.04 (0.04) | 0.63 | 0.00 (0.00) | 0.00 (0.00) | 0.77 | 0.00 (0.01) | 0.00 (0.00) | 0.675 | 0.01 (0.02) | 0.03 (0.02) | 0.066 |
| Mesangial cells | 0.03 (0.02) | 0.02 (0.02) | 0.03 | 0.07 (0.04) | 0.05 (0.04) | 0.411 | 0.01 (0.03) | 0.00 (0.01) | 0.272 | 0.01 (0.02) | 0.00 (0.01) | 0.129 |
| Monocytes | 0.04 (0.04) | 0.03 (0.04) | 0.51 | 0.06 (0.09) | 0.07 (0.05) | 0.886 | 0.05 (0.05) | 0.06 (0.05) | 0.8 | 0.05 (0.04) | 0.01 (0.02) | 0.006 |
| MPP | 0.03 (0.03) | 0.01 (0.02) | 0.04 | 0.04 (0.04) | 0.05 (0.06) | 0.87 | 0.10 (0.10) | 0.09 (0.08) | 0.631 | 0.10 (0.04) | 0.07 (0.06) | 0.152 |
| MSC | 0.14 (0.09) | 0.17 (0.09) | 0.19 | 0.04 (0.06) | 0.10 (0.12) | 0.514 | 0.18 (0.23) | 0.12 (0.12) | 0.43 | 0.08 (0.07) | 0.17 (0.07) | 0.007 |
| mv Endothelial | 0.11 (0.04) | 0.12 (0.05) | 0.56 | 0.10 (0.08) | 0.08 (0.04) | 0.63 | 0.19 (0.11) | 0.15 (0.06) | 0.285 | 0.12 (0.06) | 0.11 (0.04) | 0.667 |
| Myocytes | 0.01 (0.01) | 0.01 (0.01) | 0.06 | 0.01 (0.01) | 0.01 (0.01) | 0.568 | 0.01 (0.01) | 0.01 (0.01) | 0.066 | 0.00 (0.00) | 0.00 (0.00) | 0.984 |
| naive B-cells | 0.00 (0.01) | 0.00 (0.00) | 0.43 | 0.00 (0.00) | 0.00 (0.00) | 0.689 | 0.00 (0.00) | 0.00 (0.00) | 0.321 | 0.00 (0.00) | 0.00 (0.00) | 0.35 |
| Neurons | 0.01 (0.00) | 0.01 (0.01) | 0.19 | 0.03 (0.02) | 0.03 (0.03) | 0.847 | 0.02 (0.02) | 0.01 (0.01) | 0.393 | 0.01 (0.01) | 0.01 (0.01) | 0.005 |
| Neutrophils | 0.06 (0.03) | 0.06 (0.04) | 0.67 | 0.09 (0.13) | 0.10 (0.05) | 0.936 | 0.06 (0.04) | 0.05 (0.03) | 0.544 | 0.06 (0.03) | 0.02 (0.02) | 0.001 |
| NK cells | 0.06 (0.04) | 0.06 (0.03) | 0.48 | 0.00 (0.00) | 0.02 (0.01) | 0.141 | 0.02 (0.04) | 0.01 (0.02) | 0.234 | 0.04 (0.03) | 0.02 (0.03) | 0.309 |
| NKT | 0.11 (0.07) | 0.09 (0.06) | 0.2 | 0.12 (0.11) | 0.11 (0.12) | 0.912 | 0.20 (0.21) | 0.14 (0.09) | 0.391 | 0.05 (0.05) | 0.07 (0.05) | 0.365 |
| Osteoblast | 0.14 (0.08) | 0.12 (0.07) | 0.24 | 0.08 (0.11) | 0.12 (0.06) | 0.39 | 0.03 (0.04) | 0.03 (0.03) | 0.948 | 0.03 (0.07) | 0.12 (0.08) | 0.008 |
| pDC | 0.01 (0.01) | 0.01 (0.01) | 0.41 | 0.00 (0.00) | 0.00 (0.00) | 0.77 | 0.01 (0.01) | 0.02 (0.02) | 0.055 | 0.00 (0.00) | 0.01 (0.01) | 0.012 |

| | | | | | | | | | | | | |
|---|---|---|---|---|---|---|---|---|---|---|---|---|
| Pericytes | 0.04 (0.02) | 0.04 (0.02) | 0.72 | 0.01 (0.00) | 0.02 (0.02) | 0.262 | 0.06 (0.05) | 0.04 (0.02) | 0.29 | 0.03 (0.01) | 0.02 (0.01) | 0.013 |
| Plasma cells | 0.03 (0.03) | 0.03 (0.02) | 0.58 | 0.00 (0.00) | 0.01 (0.01) | 0.525 | 0.02 (0.02) | 0.02 (0.02) | 0.76 | 0.05 (0.03) | 0.09 (0.02) | 0.001 |
| Platelets | 0.02 (0.02) | 0.02 (0.02) | 0.71 | 0.02 (0.03) | 0.02 (0.03) | 0.801 | 0.03 (0.02) | 0.03 (0.02) | 0.905 | 0.01 (0.01) | 0.00 (0.01) | 0.057 |
| Preadipocytes | 0.05 (0.02) | 0.05 (0.02) | 0.46 | 0.05 (0.07) | 0.05 (0.03) | 0.847 | 0.02 (0.02) | 0.05 (0.03) | 0.018 | 0.03 (0.02) | 0.03 (0.01) | 0.739 |
| pro B-cells | 0.00 (0.01) | 0.01 (0.01) | 0.05 | 0.02 (0.01) | 0.02 (0.02) | 0.67 | 0.02 (0.02) | 0.02 (0.02) | 0.414 | 0.01 (0.01) | 0.00 (0.00) | 0.459 |
| Sebocytes | 0.00 (0.00) | 0.00 (0.00) | 0.01 | 0.01 (0.00) | 0.01 (0.01) | 0.82 | 0.00 (0.00) | 0.00 (0.00) | 0.236 | 0.01 (0.00) | 0.00 (0.00) | 0.001 |
| Skeletal muscle | 0.01 (0.01) | 0.01 (0.01) | 0.59 | 0.01 (0.01) | 0.02 (0.02) | 0.387 | 0.01 (0.02) | 0.01 (0.01) | 0.686 | 0.01 (0.01) | 0.01 (0.00) | 0.433 |
| Smooth muscle | 0.05 (0.04) | 0.05 (0.04) | 0.93 | 0.00 (0.00) | 0.01 (0.02) | 0.586 | 0.29 (0.14) | 0.33 (0.08) | 0.385 | 0.01 (0.02) | 0.02 (0.03) | 0.312 |
| Tgd cells | 0.05 (0.03) | 0.05 (0.03) | 0.99 | 0.00 (0.00) | 0.00 (0.01) | 0.639 | 0.02 (0.02) | 0.01 (0.02) | 0.662 | 0.02 (0.02) | 0.03 (0.02) | 0.252 |
| Th1 cells | 0.11 (0.07) | 0.10 (0.07) | 0.86 | 0.16 (0.05) | 0.18 (0.06) | 0.575 | 0.08 (0.07) | 0.07 (0.07) | 0.665 | 0.03 (0.05) | 0.08 (0.05) | 0.016 |
| Th2 cells | 0.01 (0.01) | 0.01 (0.01) | 0.09 | 0.00 (0.00) | 0.01 (0.02) | 0.555 | 0.04 (0.05) | 0.04 (0.04) | 0.982 | 0.06 (0.03) | 0.05 (0.04) | 0.708 |
| Tregs | 0.02 (0.03) | 0.02 (0.02) | 0.95 | 0.14 (0.01) | 0.11 (0.07) | 0.576 | 0.01 (0.02) | 0.01 (0.01) | 0.488 | 0.06 (0.03) | 0.04 (0.04) | 0.245 |
| ImmuneScore | 0.35 (0.11) | 0.32 (0.10) | 0.22 | 0.35 (0.14) | 0.35 (0.08) | 0.955 | 0.32 (0.13) | 0.26 (0.09) | 0.212 | 0.28 (0.09) | 0.17 (0.06) | 0.003 |
| StromaScore | 0.09 (0.04) | 0.10 (0.04) | 0.44 | 0.08 (0.05) | 0.05 (0.04) | 0.224 | 0.05 (0.04) | 0.05 (0.03) | 0.956 | 0.07 (0.03) | 0.07 (0.02) | 0.672 |
| Microenvironm | 0.44 (0.12) | 0.42 (0.12) | 0.41 | 0.43 (0.10) | 0.40 (0.08) | 0.636 | 0.37 (0.13) | 0.31 (0.10) | 0.218 | 0.35 (0.10) | 0.24 (0.06) | 0.005 |

**Supplementary Table 3.The list of primers**

| qRT-PCR primer | Sequence |
|---|---|
| ARNTL Forward Primer | TGCAACGCAATGTCCAGGAA |
| ARNTL Reverse Primer | GGTGGCACCTCTTAATGTTTTCA |
| BTG2 Forward Primer | CCTGTGGGTGGACCCCTAT |
| BTG2 Reverse Primer | GGCCTCCTCGTACAAGACG |
| CXCL10 Forward Primer | GTGGCATTCAAGGAGTACCTC |
| CXCL10 Reverse Primer | TGATGGCCTTCGATTCTGGATT |
| CCL4 Forward Primer | CTGTGCTGATCCCAGTGAATC |
| CCL4 Reverse Primer | TCAGTTCAGTTCCAGGTCATACA |
| CHI3L1 Forward Primer | AAGCAACGATCACATCGACAC |
| CHI3L1 Reverse Primer | TCAGGTTGGGGTTCCTGTTCT |
| IER3 Forward Primer | CAGCCGCAGGGTTCTCTAC |
| IER3 Reverse Primer | GATCTGGCAGAAGACGATGGT |
| FOS Forward Primer | CCGGGGATAGCCTCTCTTACT |
| FOS Reverse Primer | CCAGGTCCGTGCAGAAGTC |
| PPARGC1A Forward Primer | TCTGAGTCTGTATGGAGTGACAT |
| PPARGC1A Reverse Primer | CCAAGTCGTTCACATCTAGTTCA |
| SOCS2 Forward Primer | CAGATGTGCAAGGATAAGCGG |
| SOCS2 Reverse Primer | GCGGTTTGGTCAGATAAAGGTG |
| GAPDH Forward Primer | CTGGGCTACACTGAGCACC |
| GAPDH Reverse Primer | AAGTGGTCGTTGAGGGCAATG |

# TRIPOD Checklist: Prediction Model Development and Validation

| Section/Topic | Item | | Checklist Item | Page |
|---|---|---|---|---|
| **Title and abstract** | | | | |
| Title | 1 | D;V | Identify the study as developing and/or validating a multivariable prediction model, the target population, and the outcome to be predicted. | 1 |
| Abstract | 2 | D;V | Provide a summary of objectives, study design, setting, participants, sample size, predictors, outcome, statistical analysis, results, and conclusions. | 1 |
| **Introduction** | | | | |
| Background and objectives | 3a | D;V | Explain the medical context (including whether diagnostic or prognostic) and rationale for developing or validating the multivariable prediction model, including references to existing models. | 2 |
| | 3b | D;V | Specify the objectives, including whether the study describes the development or validation of the model or both. | 2 |
| **Methods** | | | | |
| Source of data | 4a | D;V | Describe the study design or source of data (e.g., randomized trial, cohort, or registry data), separately for the development and validation data sets, if applicable. | 2 |
| | 4b | D;V | Specify the key study dates, including start of accrual; end of accrual; and, if applicable, end of follow-up. | 2 |
| Participants | 5a | D;V | Specify key elements of the study setting (e.g., primary care, secondary care, general population) including number and location of centres. | 2 |
| | 5b | D;V | Describe eligibility criteria for participants. | 2 |
| | 5c | D;V | Give details of treatments received, if relevant. | – |
| Outcome | 6a | D;V | Clearly define the outcome that is predicted by the prediction model, including how and when assessed. | 3 |
| | 6b | D;V | Report any actions to blind assessment of the outcome to be predicted. | 3 |
| Predictors | 7a | D;V | Clearly define all predictors used in developing or validating the multivariable prediction model, including how and when they were measured. | 3 |
| | 7b | D;V | Report any actions to blind assessment of predictors for the outcome and other predictors. | – |
| Sample size | 8 | D;V | Explain how the study size was arrived at. | 2 |
| Missing data | 9 | D;V | Describe how missing data were handled (e.g., complete-case analysis, single imputation, multiple imputation) with details of any imputation method. | – |
| Statistical analysis methods | 10a | D | Describe how predictors were handled in the analyses. | 3 |
| | 10b | D | Specify type of model, all model-building procedures (including any predictor selection), and method for internal validation. | 3 |
| | 10c | V | For validation, describe how the predictions were calculated. | 3 |
| | 10d | D;V | Specify all measures used to assess model performance and, if relevant, to compare multiple models. | 3 |
| | 10e | V | Describe any model updating (e.g., recalibration) arising from the validation, if done. | – |
| Risk groups | 11 | D;V | Provide details on how risk groups were created, if done. | – |
| Development vs. validation | 12 | V | For validation, identify any differences from the development data in setting, eligibility criteria, outcome, and predictors. | – |
| **Results** | | | | |
| Participants | 13a | D;V | Describe the flow of participants through the study, including the number of participants with and without the outcome and, if applicable, a summary of the follow-up time. A diagram may be helpful. | 3-5 |
| | 13b | D;V | Describe the characteristics of the participants (basic demographics, clinical features, available predictors), including the number of participants with missing data for predictors and outcome. | 3-6 |
| | 13c | V | For validation, show a comparison with the development data of the distribution of important variables (demographics, predictors and outcome). | 6 |
| Model development | 14a | D | Specify the number of participants and outcome events in each analysis. | 6/7/9/11 |
| | 14b | D | If done, report the unadjusted association between each candidate predictor and outcome. | 13 |
| Model specification | 15a | D | Present the full prediction model to allow predictions for individuals (i.e., all regression coefficients, and model intercept or baseline survival at a given time point). | 13-14 |
| | 15b | D | Explain how to the use the prediction model. | 13-14 |
| Model performance | 16 | D;V | Report performance measures (with CIs) for the prediction model. | 13 |
| Model-updating | 17 | V | If done, report the results from any model updating (i.e., model specification, model performance). | – |
| **Discussion** | | | | |
| Limitations | 18 | D;V | Discuss any limitations of the study (such as nonrepresentative sample, few events per predictor, missing data). | 15 |
| Interpretation | 19a | V | For validation, discuss the results with reference to performance in the development data, and any other validation data. | 15 |
| | 19b | D;V | Give an overall interpretation of the results, considering objectives, limitations, results from similar studies, and other relevant evidence. | 14-15 |
| Implications | 20 | D;V | Discuss the potential clinical use of the model and implications for future research. | 15 |
| **Other information** | | | | |
| Supplementary information | 21 | D;V | Provide information about the availability of supplementary resources, such as study protocol, Web calculator, and data sets. | 16 |
| Funding | 22 | D;V | Give the source of funding and the role of the funders for the present study. | 16 |

*Items relevant only to the development of a prediction model are denoted by D, items relating solely to a validation of a prediction model are denoted by V, and items relating to both are denoted D;V. We recommend using the TRIPOD Checklist in conjunction with the TRIPOD Explanation and Elaboration document.
